# From Efficacy to Process: Uric-Acid-Lowering Effect of *Limosilactobacillus reuteri* W6-12 and Its Production Process Optimization

**DOI:** 10.3390/foods15193451

**Published:** 2026-09-27

**Authors:** Yu Hao, Yuwei Wu, Qihui Gu, Xinyu Zhao, Tingting Liang, Ying Li, Hui Zhao, Youxiong Zhang, Xianhu Wei, He Gao, Qingping Wu, Xinqiang Xie, Ling Chen

**Affiliations:** 1 College of Food Science, Northeast Agricultural University, Harbin 150006, China; haoxiaoyu1994@163.com; 2 State Key Laboratory of Applied Microbiology Southern China, Guangdong Provincial Key Laboratory of Microbial Safety and Health, National Health Commission Science and Technology Innovation Platform for Nutrition and Safety of Microbial Food, Key Laboratory of Big Data Technologies for Food Microbiological Safety, State Administration for Market Regulation, Institute of Microbiology, Guangdong Academy of Sciences, Guangzhou 510070, China; 3 Food and Drug Laboratory, Guangdong Detection Centre of Microbiology, Guangzhou 510070, China

**Keywords:** *Limosilactobacillus reuteri* W6-12, hyperuricemia, gut microbiota, bile acid metabolism, enterohepatic circulation, response surface methodology

## Abstract

Clinical chemical drugs for hyperuricemia (HUA) often cause side effects including liver and kidney injury. Developing safe and effective probiotic preparations for HUA intervention is of considerable application value. However, the large-scale production of probiotics faces two major bottlenecks: low viable cell density during fermentation and poor survival after lyophilization. This study integrated in vivo efficacy evaluation and mechanism exploration with the production process optimization for *Limosilactobacillus reuteri* W6-12. We first verified its hypouricemic effect in vivo, then adopted response surface methodology to optimize fermentation medium components and composite cryoprotectant formulations, and further evaluated the efficacy of the obtained lyophilized probiotic powder using a rat model of HUA. In HUA rats, W6-12 treatment significantly decreased serum UA (SUA) levels, an effect attributed to hepatic xanthine oxidase inhibition and renal downregulation of GLUT9 and URAT1, alleviation of kidney oxidative stress and inflammation, and reshaping of gut microbial composition and associated metabolites, likely via the gut microbiota-bile acid axis. The optimized medium (sucrose 31 g/L, yeast extract 26 g/L, sodium acetate 7.5 g/L) yielded 2.03 × 10^9^ CFU/mL, a 1.6-fold increase over the unoptimized condition. The optimal composite cryoprotectant consisted of 8% L-sodium glutamate, 10% trehalose, and 12% skim milk powder, achieving a lyophilization survival rate of 89.47%. Low, medium, and high doses of lyophilized W6-12 all exerted dose-dependent urate-lowering effects in HUA rats. In conclusion, the lyophilized powder of *L. reuteri* W6-12 is a promising probiotic preparation for UA management.

## 1. Introduction

Hyperuricemia (HUA) is a chronic metabolic disorder driven by purine metabolism dysfunction, characterized by abnormally high serum uric acid (SUA) levels [1]. The global prevalence of HUA has been rising steadily, ranking as the fourth most common chronic metabolic disease after hypertension, hyperlipidemia, and hyperglycemia. HUA imposes a heavy and persistent public health burden, with its morbidity rate second only to diabetes [2]. Clinical and epidemiological evidence has shown that HUA is not only a key pathogenic factor in gout and gouty arthritis but also a contributor to multiple complications. Metabolic disorders, oxidative stress, and inflammatory imbalance induced by persistent HUA further accelerate the progression of chronic kidney disease, cardiovascular damage, and type 2 diabetes [3,4]. At present, clinical interventions for HUA primarily depend on xanthine oxidase (XOD) inhibitors and uricosuric agents, typically represented by allopurinol, febuxostat, and benzbromarone. Although these pharmaceuticals possess definite uric-acid-lowering efficacy, prolonged administration can lead to adverse effects such as hepatic and renal injury, allergic reactions, and drug tolerance. Additionally, clinical intolerance in some patients necessitates treatment discontinuation, which greatly limits the applicable population of conventional HUA therapies [3,5]. In this context, the exploration of safe, mild, and long-term dietary intervention strategies for HUA has become a research hotspot in the fields of functional food development and metabolic nutrition. Probiotics possess superior biosafety and multi-target metabolic regulatory properties, which render them ideal dietary functional factors for improving UA metabolism and ameliorating systemic metabolic disorders [6,7].

*L. reuteri*, a lactic acid bacterium with probiotic properties, can stably inhabit the gut of humans and animals. It is listed in China’s List of Bacterial Strains Available for Food and internationally recognized as a safe food-grade probiotic, with broad applications in fermented food manufacturing and functional dietary supplementation [8]. Multiple studies have verified that *L. reuteri* alleviates HUA symptoms by modulating purine metabolism through diverse pathways, including the degradation of purine nucleoside precursors (inosine and guanosine), suppression of XOD activity, remodeling of dysregulated gut microbiota, and maintenance of intestinal metabolic homeostasis [9,10]. Notably, the functional properties of probiotics are strictly strain-specific. Variations in strain origin, genotype, and physiological characteristics lead to distinct differences in purine degradation capability, stress tolerance, and in vivo metabolic regulatory mechanisms. In a study by Hussain et al. [11], *L. reuteri* HCS02-001 was found to remodel intestinal microbiota homeostasis, resulting in activation of the TLR4/MyD88/NF-κB signaling axis, thereby regulating UA synthesis and excretion and relieving hyperuricemic injury in mice. Zhao et al. [3] further demonstrated that *L. reuteri* NCUH064056 possessed prominent nucleoside degradation activity and XOD inhibitory capacity. These findings confirm the promising application potential of *L. reuteri* in UA reduction. Nevertheless, considerable functional heterogeneity exists among different *L. reuteri* strains, such that existing research conclusions cannot be universally extended to newly isolated strains. The in vivo intervention efficacy and intrinsic molecular mechanisms of specific strains require further systematic exploration and verification.

Current global research on uric-acid-lowering probiotics predominantly focuses on strain resource excavation, in vitro functional evaluation, and preliminary investigation of in vivo regulatory mechanisms. However, insufficient attention has been paid to translational research bridging laboratory functional verification and large-scale industrial production, which constitutes a major technical bottleneck restricting the industrialization of uric-acid-lowering probiotic products. High-density liquid fermentation is an indispensable procedure for low-cost, large-scale industrial production of probiotics. Conventional MRS medium is inadequate for industrial fermentation due to its high raw material cost and unstable batch fermentation performance. Response surface methodology-mediated medium optimization has been validated as an efficient strategy to enhance viable cell yield and reduce production costs [12,13]. Vacuum freeze-drying is a core processing technique for the commercial preservation and application of lyophilized probiotic powder, which effectively maintains bacterial cellular activity. Nonetheless, ice crystal formation and sustained water loss during lyophilization disrupt cell membrane integrity and trigger irreversible denaturation of intracellular proteins, substantially reducing bacterial survival rate and impairing the functional stability of probiotics [14,15]. A well-formulated composite cryoprotectant system can effectively mitigate freeze-drying-induced cellular stress and is critical for the fabrication of high-quality lyophilized probiotic powder. At present, most relevant studies primarily focus on improving lyophilization survival rate, while neglecting the potential impact of freeze-drying procedures on the core uric-acid-lowering function of target strains. Furthermore, fermentation medium components directly modulate the fatty acid profile of bacterial cell membranes and the accumulation of intracellular compatible solutes, thereby altering the stress resistance of probiotic strains. This evidence indicates a tight coupling relationship between fermentation and lyophilization processes [16]. Accordingly, individual optimization of a single processing step fails to simultaneously achieve high viable cell productivity and stable functional retention of probiotics. Integrated and systematic optimization of fermentation medium formulas and composite cryoprotectant systems is therefore essential for the industrial preparation of high-activity uric-acid-lowering lyophilized probiotic powder.

Our research group previously isolated and screened a strain of *L. reuteri* W6-12 from traditional Tibetan milk residue. In vitro experiments confirmed that this strain could efficiently degrade purine nucleosides including inosine, guanosine, and adenosine [17], indicating its potential for uric-acid-lowering activity (patent No.: 202610366793.1). However, its in vivo uric-acid-lowering efficacy, regulatory mechanisms underlying gut microbiota and metabolic network modulation, and functional stability following large-scale fermentation and freeze-drying remain unclear. To address these research gaps, the present study first established a rat model of HUA to systematically evaluate the in vivo uric-acid-lowering capacity and renal protective effects of strain W6-12. 16S rRNA sequencing coupled with serum and fecal untargeted metabolomics was subsequently employed to elucidate the strain-mediated regulatory network between gut microbiota and metabolites. On this basis, optimization of the fermentation medium and composite cryoprotectant formulations was conducted using response surface methodology, aiming to establish a high-density, high-survival-rate lyophilized probiotic powder preparation process with potential for further scale-up. Finally, gradient doses of lyophilized probiotic powder were administered to HUA model rats to verify the dose-response relationship of its uric-acid-lowering effects and evaluate the functional retention of the strain after fermentation and lyophilization. This study aims to construct a complete technical system covering strain functional verification, process optimization, and finished product efficacy evaluation, providing reliable experimental evidence and technical references for the further development of functional foods supplemented with uric-acid-lowering probiotics.

## 2. Materials and Methods

### 2.1. Materials and Reagents

*L. reuteri* W6-12, previously isolated and screened by our research group from traditional Tibetan milk residue. The strain was deposited at the Guangdong Microbial Culture Collection Center (accession number: GDCC 67938).

Basic reagents and medium components included glucose, sucrose, lactose, trehalose, isomaltose, yeast extract, sodium acetate (CH_3_COONa), dipotassium hydrogen phosphate (K_2_HPO_4_), diammonium hydrogen citrate (C_6_H_14_N_2_O_7_), magnesium sulfate (MgSO_4_), manganese sulfate (MnSO_4_), inulin, Tween-80, whey protein powder, soybean peptone, and food-grade MRS medium (Guangdong Huankai Microbial Technology Co., Ltd., Guangzhou, China). L-Sodium glutamate (analytical grade) was purchased from Shanghai Macklin Biochemical Technology Co., Ltd., Shanghai, China; maltitol (analytical grade) from Shanghai Dibai Biotechnology Co., Ltd., Shanghai, China; xylooligosaccharide, fructooligosaccharide, resistant dextrin and maltodextrin (analytical grade) from Shanghai Yuanye Biotechnology Co., Ltd., Shanghai, China; peanut meal, cottonseed meal, corn steep liquor and chitosan oligosaccharide (analytical grade) from Beijing Hongrun Baoshun Technology Co., Ltd., Beijing, China; food-grade skim milk powder was obtained from Beidahuang Wondersun Dairy Co., Ltd., Harbin, China. All reagents and raw materials met the standards for analytical testing and food-grade application.

### 2.2. Strain Activation and Seed Culture Preparation

The glycerol-preserved stock of *L. reuteri* W6-12 was thawed at room temperature and inoculated into sterile liquid MRS medium at 2% (*v*/*v*) inoculation volume. For primary activation, the culture was shaken at 100 r/min and 37 °C for a period of 18–24 h. To restore strain physiological activity, 2–3 continuous subcultures were conducted until the culture reached logarithmic growth phase, which was defined as seed culture for subsequent experiments. Cell viability was assessed by the pour plate method, and bacterial counts were expressed in CFU/mL.

### 2.3. Establishment of Hyperuricemic Rat Model and Bacterial Suspension Intervention

#### 2.3.1. Experimental Animals

SPF-grade male SD rats (initial body weight: 180–200 g) were obtained from the Guangdong Medical Laboratory Animal Center. They were maintained in the animal facility at the Institute of Microbiology, Guangdong Academy of Sciences (Science City) under controlled conditions (temperature: 20–26 °C, relative humidity: 40–70%, 12 h light/dark cycle). Standard chow and water were provided ad libitum. After one week of acclimatization, the experiments commenced. All procedures involving animals were approved by the Animal Care and Use Committee of the Institute of Microbiology, Guangdong Academy of Sciences (approval No. GT-IACUC202502063) and were performed in strict accordance with the Guide for the Care and Use of Laboratory Animals.

#### 2.3.2. Model Establishment and Group Intervention

Following acclimatization, the rats were randomly assigned to four groups (*n* = 6 per group): control (CON), hyperuricemia model (HUA), W6-12 intervention (W6-12), and allopurinol positive control (ALL). To establish the HUA model, rats in the HUA, W6-12, and ALL groups were given a daily intragastric gavage of mixed suspension containing potassium oxonate (750 mg/kg) and hypoxanthine (100 mg/kg) dissolved in 0.5% CMC-Na solution over a period of four weeks. Rats in the CON group received an equal volume of 0.5% CMC-Na by gavage. One hour after model induction, the W6-12 group was treated with freshly prepared W6-12 bacterial suspension (1 × 10^9^ CFU/kg BW), the ALL group received allopurinol solution (3 mg/kg BW), whereas the CON and HUA groups received the same volume of sterile saline via gavage. All intervention treatments lasted 4 weeks. Body weight was monitored weekly, and the gavage volume was adjusted dynamically according to weight changes. The CON and HUA groups served as the comparison baselines. In the figures, statistical significance between the HUA and CON groups is indicated by ^#^, while * denotes significance between treatment groups and the HUA group.

### 2.4. Sample Collection and Biochemical Index Detection

#### 2.4.1. Sample Collection

After the last intervention, all rats were fasted for 12 h with water freely available. Animals were anesthetized by intramuscular injection of Zoletil (tiletamine-zolazepam, 40 mg/kg BW), and blood was drawn from the abdominal aorta. Blood samples were left to stand at room temperature for 2 h and then centrifuged at 3000× *g* and 4 °C for 15 min to obtain serum, which was collected and stored at −80 °C for subsequent analyses. After exsanguination, the rats were euthanized, and the liver and kidneys were rapidly excised, rinsed with sterile saline to remove residual blood, and processed accordingly. Portions of kidney tissue were fixed in 4% paraformaldehyde for histopathological examination, while the remaining tissues were snap-frozen in liquid nitrogen and maintained at −80 °C for biochemical assays. Fresh fecal samples were collected aseptically into sterile EP tubes and preserved at −80 °C for 16S rRNA sequencing and untargeted metabolomics.

#### 2.4.2. Biochemical Indicator Assays

Serum UA, creatinine (CRE) and blood urea nitrogen (BUN) were quantified with commercial kits per the manufacturer’s protocols (Nanjing Jiancheng Bioengineering Institute, Nanjing, China). Hepatic XOD and adenosine deaminase (ADA) activities were detected simultaneously. Renal GLUT9, URAT1 and ROS levels were quantified using kits from Jiangsu Bosheng Biotechnology Co., Ltd. (Nanjing, China), and renal IL-6, IL-1β and MDA levels were determined with kits supplied by Beijing Dongge Boya Biotechnology Co., Ltd. (Beijing, China).

#### 2.4.3. Renal Histopathological Examination

Paraformaldehyde-fixed (4%) kidney samples underwent gradient dehydration, paraffin embedding, and serial sectioning. The resulting sections were then subjected to hematoxylin and eosin (HE) and Masson’s trichrome staining, followed by dehydration, transparency and mounting with neutral balsam. Each group was assessed for renal tubular integrity, epithelial cell morphology, and inflammatory infiltration. Images were captured under a light microscope.

### 2.5. 16S rRNA High-Throughput Sequencing and Metabolomics Analysis

Fecal samples from rats were subjected to 16S rRNA high-throughput sequencing by Shanghai Majorbio Biomedical Technology Co., Ltd., Shanghai, China. Metabolites in serum and fecal samples were extracted using methanol–acetonitrile mixed solvent, after centrifugation, the resulting supernatants were subjected to untargeted metabolomics analysis on a UPLC-MS/MS platform. Data acquisition was performed in positive and negative ionization modes. Peak detection was carried out on the raw data, peak alignment and normalization, followed by metabolite annotation and qualitative and quantitative analysis based on the Human Metabolome Database (HMDB). Metabolites with VIP > 1, |log_2_FC| ≥ 1 and unadjusted *p* < 0.05 were considered differentially abundant. As the untargeted metabolomics analysis was exploratory, no multiple-testing correction was applied, and the identified metabolites should be regarded as candidates requiring further validation.

### 2.6. Fermentation Medium Optimization

#### 2.6.1. Single-Factor and Plackett-Burman Experiments

The basal medium was composed of glucose (20 g/L), yeast extract (18 g/L), K_2_HPO_4_ (2 g/L), CH_3_COONa (5 g/L), ammonium sulfate (2 g/L), MnSO_4_ (0.05 g/L), MgSO_4_ (0.2 g/L), and Tween-80 (1 g/L). To assess the effects of carbon sources, nitrogen sources, buffer salts, trace elements, and surfactants on the growth of *L. reuteri* W6-12, viable cell count was employed as the evaluation metric.

Single-factor variable settings were as follows: carbon sources (glucose, sucrose, isomaltose, trehalose, lactose) were fixed at 20 g/L, and concentration gradients of 10, 15, 20, 25, 30 and 35 g/L were set for the optimal carbon source; nitrogen sources (corn steep liquor powder, yeast extract, soybean peptone, peanut meal, cottonseed meal) were fixed at 20 g/L, with concentration gradients of 18, 22, 26, 30 and 34 g/L for the optimal nitrogen source; gradient concentrations were set for buffer salts (C_6_H_14_N_2_O_7_ (0–4 g/L), K_2_HPO_4_ (0–4 g/L), anhydrous CH_3_COONa (0–10 g/L)), trace elements (MgSO_4_ (0–0.4 g/L), MnSO_4_ (0–0.1 g/L)), and Tween-80 (0–2 g/L). A single-factor control principle was adopted, where only one factor was altered in each experiment while other medium components and culture conditions remained unchanged. This allowed the effect of each component to be evaluated independently and defined the factor ranges used in the subsequent Box-Behnken design. Seed cultures were inoculated at 2% into 5 mL liquid medium and anaerobically cultured at 37 °C for 24 h. Viable cell counts were measured by the pour plate method to screen the optimal type and concentration of each factor.

Following the single-factor experiments, a Plackett-Burman design with eight factors, namely sucrose, yeast extract, K_2_HPO_4_, C_6_H_14_N_2_O_7_, CH_3_COONa, MgSO_4_, MnSO_4_, and Tween-80, was performed in 12 runs, using viable cell count as the response. The significant factors identified were subsequently optimized using a Box-Behnken design.

#### 2.6.2. Box-Behnken Design for Fermentation Medium Optimization

Based on the results of the single-factor and Plackett-Burman experiments, the three most significant factors affecting W6-12 proliferation were selected for further optimization. These were further optimized using a Box-Behnken design (three factors, three levels). A Box-Behnken design was selected because it avoids experimental runs at extreme factor combinations, which may produce unstable samples in this system, and because the goal was to model the response within the pre-defined factor ranges rather than to extrapolate beyond them. The optimal concentration of each factor obtained from single-factor tests was set as the central point (0 level), with high (+1) and low (−1) levels established accordingly. Using viable cell count as the response, 17 experimental runs were generated and analyzed via Design-Expert 13.0 software. The optimal medium formulation was determined by fitting a quadratic polynomial regression model to the data.

#### 2.6.3. Verification of Fermentation Medium Optimization

Three parallel fermentation validation trials were performed using the optimal medium formula predicted by the response surface model. Actual viable cell counts were determined and compared with model predicted values to verify the reliability of the fitted model and the robustness of the optimal medium composition.

### 2.7. Lyophilization Cryoprotectant Optimization

#### 2.7.1. Single-Factor Screening Experiments

Sterile PBS buffer was used as blank control. A total of 17 candidate cryoprotectants were selected, including L-sodium glutamate, chitosan oligosaccharide, L-glutamic acid, mannitol, maltitol, ascorbic acid, isomaltooligosaccharide, fructooligosaccharide, maltodextrin, resistant dextrin, trehalose, whey protein, inulin, L-glutamine, L-tryptophan, skim milk powder and L-lysine. Each cryoprotectant was tested at a mass concentration of 10% (*w*/*v*), with the volume ratio of bacterial pellet to cryoprotectant solution fixed at 1:4. After lyophilization, viable cell counts before and after freeze-drying were detected to calculate survival rate, so as to screen out three cryoprotectants with superior protective performance.

Lyophilization survival rate (%) = (CFU/mL post-lyophilization/CFU/mL pre-lyophilization) × 100%

On the basis of primary screening results, gradient mass concentrations were set for single-factor experiments to further explore the influence of each cryoprotectant concentration on the lyophilization survival rate of W6-12 and confirm the optimal dosage of each cryoprotectant.

#### 2.7.2. Box-Behnken Design for Cryoprotectant Formulation Optimization

Three cryoprotectants with outstanding protective effects (trehalose, L-sodium glutamate and skim milk powder) were screened from single-factor results for three-factor three-level Box-Behnken optimization. Lyophilization survival rate was set as the response value to analyze the interactive effects among the three components and determine the optimal composite cryoprotectant formula.

#### 2.7.3. Verification of Cryoprotectant Formulation Optimization

Three parallel lyophilization replicates were conducted using the optimized composite cryoprotectant formula. Actual lyophilization survival rates were measured and compared with model predicted values to validate the stability of composite protectant formula and the accuracy of the optimization model.

### 2.8. Preparation of Lyophilized Probiotic Powder

High-density fermentation of *L. reuteri* W6-12 was performed using the optimal fermentation medium described in Section 2.6, combined with the optimal composite cryoprotectant system screened in Section 2.7. lyophilized probiotic powder was prepared following standardized lyophilization procedures. The viable cell count of finished powder was quantified via the pour plate method, and qualified powder was stored for subsequent animal efficacy verification experiments.

### 2.9. In Vivo Efficacy Verification of Lyophilized Probiotic Powder

Another set of SPF-grade male SD rats underwent one week of acclimation and were then randomized into six groups (six rats each): CON (blank control), HUA model, W6-12-L (low dose, 1 × 10^8^ CFU/kg BW), W6-12-M (medium dose, 1 × 10^9^ CFU/kg BW), W6-12-H (high dose, 1 × 10^10^ CFU/kg BW), and ALL (allopurinol, 3 mg/kg BW). The HUA induction procedure was consistent with Section 2.3.2. All animal procedures for this experiment were approved by the Animal Care and Use Committee of the Institute of Microbiology, Guangdong Academy of Sciences (approval No. GT-IACUC202512045). Each intervention group received daily intragastric administration of W6-12 powder suspension in normal saline at corresponding dosages for four consecutive weeks. Serum and kidney samples were harvested at the end of the study as per Section 2.4.1. Serum UA, CRE and BUN levels were detected following Section 2.4.2, and renal HE histopathological staining was conducted according to Section 2.4.3.

### 2.10. Statistical Analysis

All data were expressed as mean ± standard deviation (SD). For the animal experiments, each group consisted of six rats (*n* = 6), and intergroup differences were assessed using one-way analysis of variance (ANOVA) followed by Tukey’s multiple comparison test in GraphPad Prism 10.0. For the optimization of culture medium and lyophilized protectant, each experiment was performed in triplicate (*n* = 3). The optimization data were analyzed by quadratic multiple regression and ANOVA using Design-Expert 13.0 software, and further statistically processed using SPSS 26.0. For untargeted metabolomics data analysis, including statistical processing, differential metabolite screening, and visualization, MetaboAnalyst (https://www.metaboanalyst.ca (accessed on 26 June 2026)) and R software (version 4.4.1) with the ggplot2 and dplyr packages were used. The 16S rRNA sequencing data were analyzed using the online platform of Majorbio. A value of *p* < 0.05 was considered statistically significant.

## 3. Results and Analysis

### 3.1. Effects of Limosilactobacillus reuteri W6-12 in Hyperuricemic Rats

#### 3.1.1. Effects of W6-12 on SUA Levels and Renal Function in Hyperuricemic Rats

After oral gavage with potassium oxonate and hypoxanthine, HUA rats exhibited significantly higher SUA levels than the CON group (Figure 1A), indicating successful establishment of the HUA rat model. Following four weeks of intervention via intragastric administration of W6-12, the elevated SUA levels were significantly reversed compared with the HUA group, indicating the in vivo hypouricemic effect of *L. reuteri* W6-12. This observation aligned with previous studies reporting the hypouricemic effects of *L. reuteri* RE225 and HCS02-001 in hyperuricemic animal models [11,18].

Renal functional biomarkers are illustrated in Figure 1B,C. The HUA group exhibited markedly increased serum CRE and BUN levels relative to the CON group, demonstrating that sustained HUA induced severe renal dysfunction. In contrast, W6-12 supplementation effectively decreased serum CRE and BUN contents, suggesting that W6-12 could ameliorate hyperuricemia-triggered renal injury. Consistent with these findings, Yang et al. reported that *L. reuteri* intervention alleviated acute kidney injury by reducing serum CRE and BUN concentrations in mice [19]. Additionally, Miao et al. verified that Lacticaseibacillus rhamnosus JY027 improved abnormal renal biochemical indexes in hyperuricemic mice [20], further consolidating the protective potential of probiotics against hyperuricemia-associated renal damage.

Renal histopathological staining analyses further validated the renal protective properties of W6-12 (Figure 1D–I). HE staining results showed complete renal tubular structure and regularly arranged tubular epithelial cells in the CON group. However, the HUA group displayed typical pathological lesions, including obvious tubular vacuolar degeneration, tubular dilatation, and extensive inflammatory cell infiltration, consistent with the characteristic renal pathological changes of HUA reported previously [21]. Notably, W6-12 treatment substantially restored the structural integrity of renal tubules and remarkably relieved renal pathological damage and inflammatory infiltration. Masson staining revealed prominent interstitial collagen deposition and fibrotic lesions in the HUA group, whereas W6-12 supplementation significantly reduced collagen accumulation and inhibited renal fibrosis progression. Collectively, these results indicated that W6-12 exerted multifaceted renal protective effects by alleviating tubular epithelial damage, suppressing renal inflammatory responses, and restraining interstitial fibrosis under hyperuricemic conditions.

#### 3.1.2. Effects of W6-12 on Hepatic Purine Metabolic Enzyme Activities and Renal Urate Transporter Levels

Hepatic XOD and ADA activities were measured to elucidate how W6-12 lowers UA. XOD is the primary rate-limiting enzyme that drives the conversion of hypoxanthine and xanthine into UA, directly determining endogenous UA production. Clinically, allopurinol and febuxostat exert their uric-acid-lowering effects by targeting and inhibiting this enzyme [22]. Figure 2A showed that hepatic XOD activity was significantly elevated in the HUA group compared with the CON group, and this increase was attenuated by W6-12. Regarding ADA activity (Figure 2B), the HUA group exhibited significantly higher levels than the CON group. Although W6-12 treatment produced a reduction, the effect was not statistically significant *(p* > 0.05), indicating that W6-12 primarily regulated endogenous UA synthesis through targeted inhibition of XOD activity. Similar mechanisms have been reported for other probiotic strains. For instance, *L. reuteri* Urob-7 decreased hepatic XOD activity by 37.6% in vivo, while *L. reuteri* RE225 showed dose-dependent XOD suppression. *L. reuteri* CCFM1132 significantly reduced XOD activity in a human randomized controlled trial, and *Lactiplantibacillus plantarum* AR342 lowered SUA in hyperuricemic mice via XOD and PNP inhibition [11,23,24,25], further supporting that XOD inhibition is a conserved pathway among *Lactobacillus* strains for alleviating HUA.

The kidney is responsible for approximately two-thirds of UA excretion in the body [18]. Renal UA excretion disorder is a major contributor to the development of HUA. URAT1 and GLUT9 are core transporter proteins responsible for urate reabsorption in the renal proximal tubules, and their aberrant overexpression enhances urate reabsorption and reduces excretion [22]. Figure 2C,D revealed that the HUA group exhibited significantly higher renal GLUT9 and URAT1 protein levels than the CON group. Following W6-12 intervention, both GLUT9 and URAT1 protein levels were significantly reduced, indicating that tubular urate reabsorption was suppressed and UA excretion was promoted. In summary, W6-12 exerted its uric-acid-lowering effect through two synergistic pathways: inhibition of hepatic UA synthesis and blockade of renal urate reabsorption.

#### 3.1.3. Effects of W6-12 on Renal Oxidative Stress and Inflammatory Cytokine Levels

Hyperuricemia-induced renal injury arises from the interactive pathological cascade of oxidative stress and inflammatory responses. To clarify the mechanism underlying the renoprotective action of W6-12, the levels of key oxidative stress markers and pro-inflammatory cytokines in renal tissues were determined. As shown in Figure 2E,F, the HUA group exhibited markedly higher renal ROS and MDA levels than the CON group, demonstrating that HUA successfully triggered renal oxidative stress damage. However, W6-12 intervention significantly decreased the accumulation of renal ROS and MDA, effectively ameliorating oxidative stress lesions. This observation corroborated previous reports demonstrating that HUA promotes both mitochondrial ROS overproduction [26] and NLRP3 inflammasome activation [27], whereas probiotic supplementation can efficiently reverse hyperuricemia-related oxidative renal impairment [26,28].

Renal inflammatory cytokine analysis yielded consistent results (Figure 2G,H). The HUA group exhibited significantly elevated renal IL-6 and IL-1β concentrations, indicating that sustained HUA activated local renal inflammatory reactions. By contrast, W6-12 treatment remarkably reduced the abundances of these two pro-inflammatory factors. These results are supported by previous mechanistic studies of *L. reuteri* strains. Previous findings demonstrated that *L. reuteri* Urob-7 inhibited the NF-κB/NLRP3 inflammatory axis, with renal NF-κB and NLRP3 levels decreased by 39.3% and 47.6%, respectively [25]. Similarly, the renal TLR4/NF-κB signaling pathway was suppressed by *L. reuteri* RE225, thereby attenuating inflammatory infiltration in the kidney [23]. Moreover, *L. plantarum* MPB-65 combined with epicatechin was verified to alleviate renal inflammatory damage in hyperuricemic mice [29], further confirming the universal renal anti-inflammatory capacity of probiotics. Collectively, W6-12 intervention could simultaneously relieve renal oxidative stress and suppress local inflammatory responses, breaking the vicious cycle between oxidative damage and inflammation, and ultimately improving hyperuricemia-caused renal injury.

### 3.2. Effects of Limosilactobacillus reuteri W6-12 on Gut Microbiota Structure in HUA Rats

To explore the modulatory effect of W6-12 on intestinal microecology in hyperuricemic rats, 16S rRNA high-throughput sequencing was performed to systematically analyze gut microbial diversity, species composition, and differential microbial profiles. Microbial species richness and community evenness were evaluated using alpha diversity indices. As illustrated in Supplementary Figure S1A–C, the Chao1, ACE, and Sobs indices were markedly decreased in the HUA group and showed no significant recovery after W6-12 intervention, indicating that hyperuricemic stress triggered irreversible loss of intestinal microbial richness. Meanwhile, the HUA group exhibited a lower Shannon index and a higher Simpson index (Figure 3A,B), demonstrating that HUA simplified gut microbial community structure and facilitated the overdominance of specific bacterial taxa. These alterations were consistent with clinical cohort studies reporting reduced gut microbial alpha diversity in hyperuricemic populations [30,31]. Importantly, W6-12 treatment restored the Shannon index to nearly normal levels and reduced the Simpson index correspondingly. These results indicated that W6-12 effectively improved gut microbial community evenness, despite its limited capacity to restore overall microbial species richness.

Principal component analysis (PCA) was further used to assess beta diversity and characterize overall variations in microbial community structure. Clear spatial separation was observed among the CON, HUA, and W6-12 groups along the principal coordinate axes (Figure 3C), indicating that HUA substantially disrupted gut microbial homeostasis, while W6-12 intervention efficiently reversed such microbial dysbiosis. Venn diagram analysis visualized the shared and unique microbial genera across groups (Supplementary Figure S1D). The CON group harbored 16 unique genera, whereas the HUA group retained only 4, suggesting that HUA drastically depleted indigenous microbial species. This trend was consistent with the reduced Chao1, ACE, and Sobs indices in the HUA group. After W6-12 supplementation, the number of unique genera recovered to 14, confirming the effective reconstruction of gut microbial diversity.

Phylum-level taxonomic analysis revealed that *Firmicutes* and *Bacteroidetes* dominated the intestinal microbial community across all groups (Figure 3D). The HUA group exhibited a decreased *Firmicutes*/*Bacteroidetes* (F/B) ratio (CON: 3.89 vs. HUA: 2.60), which was consistent with previous HUA and gout studies demonstrating depleted *Firmicutes* and enriched *Bacteroidetes* under hyperuricemic conditions [32]. Compared with the other two groups, the HUA group exhibited a significantly higher relative abundance of *Actinobacteria*, which was reduced to the lowest level by W6-12 intervention. Furthermore, the abundance of *Verrucomicrobia* increased progressively from the CON and HUA groups to the W6-12 group, which was consistent with the dynamic variation of the genus *Akkermansia* at the genus level.

Genus-level microbial profiling further revealed distinct microbial dysbiosis in the HUA group (Figure 3E). Specifically, the relative abundances of *Lactobacillus* and *Bifidobacterium* were significantly upregulated in HUA rats relative to the CON group, while the abundances of key butyrate-producing bacteria, including *Ruminococcus*, *NK4A214_group*, and *norank_f__[Eubacterium]_coprostanoligenes_group*, were markedly downregulated. The depletion of butyrate-producing *Ruminococcus* observed herein aligned with previous findings in multiple hyperuricemic animal models [33].

LEfSe analysis (LDA > 2, *p* < 0.05) and Circos visualization were performed to identify signature differential taxa (Supplementary Figure S1E,F), and multi-group differential comparison was also performed (Figure 3F). Five bacterial genera, including *Lactobacillus*, *Bifidobacterium*, *[Eubacterium]_xylanophilum_group*, *Lachnospiraceae_UCG-006*, and *Coriobacteriaceae_UCG-002*, were significantly enriched in the HUA group. The synchronous enrichment of these taxa represented a typical microbial feature of hyperuricemia-associated intestinal dysbiosis. Notably, although the total abundance of *Lactobacillus* increased in the HUA group, the genus *Ligilactobacillus* peaked in the W6-12 group, followed by the HUA group and the CON group. Clinical evidence has demonstrated that *Ligilactobacillus salivarius* and other *Ligilactobacillus* members possess purine-degrading capability and exert hypouricemic effects in hyperuricemic patients [34]. Accordingly, the enrichment of *Ligilactobacillus* in the W6-12 group was attributable to W6-12-mediated microecological remodeling, rather than direct colonization of exogenous strains.

Following W6-12 intervention, the abnormally elevated abundance of *Lactobacillus* in the HUA group was effectively reversed, while the depleted butyrate-producing genera *Ruminococcus*, *NK4A214_group*, and *norank_f__[Eubacterium]_coprostanoligenes_group* were significantly restored. Collectively, these findings confirmed that W6-12 remodeled the disordered intestinal microecology of hyperuricemic rats through multifaceted regulation. W6-12 reduced the abnormally elevated abundance of *Lactobacillus* under hyperuricemic stress and selectively enriched *Ligilactobacillus*, a genus with reported purine-degrading potential. The recovery of butyrate-producing flora indicated improved intestinal barrier integrity and enhanced intestinal anti-inflammatory capacity. Such microbial remodeling strongly correlated with the alleviated renal oxidative stress and inflammation described in Section 3.1.3, demonstrating that W6-12-mediated gut microbiota improvement ameliorated hyperuricemia-induced renal injury via the gut–kidney axis.

### 3.3. Effects of Limosilactobacillus reuteri W6-12 on Serum and Fecal Metabolites in HUA Rats

PCA of the serum metabolome (Figure 4A) revealed clear separation among the CON, HUA, and W6-12 groups, indicating significant differences in metabolic profiles. Using VIP > 1, *p* < 0.05, and |log_2_FC| ≥ 1 as screening criteria, serum metabolomic analysis identified 463 differential metabolites (432 upregulated, 31 downregulated) between the HUA and CON groups (Figure 4B). These metabolites were mainly enriched in lysine biosynthesis, arginine biosynthesis, phenylalanine biosynthesis, starch and sucrose metabolism, and histidine metabolism (Figure 4C). Compared with the HUA group (Figure 4D), W6-12 intervention yielded 560 differential metabolites (111 upregulated, 449 downregulated), with enriched pathways including lysine metabolism, primary bile acid (BA) biosynthesis, lysine biosynthesis, unsaturated fatty acid biosynthesis, and steroid hormone biosynthesis (Figure 4E).

Fecal metabolomic PCA results were consistent with those of the serum metabolome (Figure 4F). Compared with the CON group (Supplementary Figure S2A), 1705 differential metabolites (492 upregulated, 1213 downregulated) were identified in the HUA group, with core enriched pathways including nucleotide metabolism, steroid biosynthesis, purine metabolism, starch and sucrose metabolism, and the pentose phosphate pathway (Supplementary Figure S2B). After W6-12 intervention (Supplementary Figure S2C), 582 differential metabolites (263 upregulated, 319 downregulated) were identified, mainly enriched in starch and sucrose metabolism, ethylbenzene degradation, methane metabolism, caprolactam degradation, and purine metabolism (Supplementary Figure S2D). These results indicated that W6-12 could target and repair intestinal metabolic disorders disrupted by HUA.

#### 3.3.1. Effects of W6-12 on Bile Acid Enterohepatic Circulation

Disrupted BA enterohepatic circulation has been increasingly recognized as a contributor to HUA progression. The present study focused on the differential profiles of serum and fecal BAs among groups (Figure 4G). The results showed that serum levels of the primary BAs cholic acid (CA) and chenodeoxycholic acid (CDCA) were elevated in the HUA group, whereas their fecal levels were decreased, presenting a typical pattern of serum accumulation and insufficient fecal excretion that signified enterohepatic circulation stasis. This observation was consistent with enhanced reabsorption of intestinal primary BAs and impaired BA excretion under HUA. After W6-12 intervention, serum CA and CDCA levels declined while fecal CA and CDCA levels increased, suggesting a restoration of the normal excretion rhythm of primary BAs.

For conjugated primary BAs, serum levels of taurochenodeoxycholic acid (TCDCA), glycochenodeoxycholic acid (GCDCA), taurodeoxycholic acid (TDCA), and glycodeoxycholic acid (GDCA) were significantly elevated in the HUA group and markedly reduced after W6-12 intervention. In contrast, serum taurocholic acid (TCA) was decreased in the HUA group and significantly upregulated following W6-12 treatment. In terms of secondary BAs, fecal deoxycholic acid (DCA) was decreased in the HUA group and further reduced after W6-12 intervention. Combined with the significant elevation of fecal CA in the W6-12 group, these findings suggested that W6-12-mediated gut microbiota remodeling altered BA conversion flux. Specifically, a large proportion of CA appeared to be excreted in its free form, which may reduce the substrate available for microbial 7α-dehydroxylation and could thereby contribute to the lower DCA production observed. These results further supported the possibility that W6-12 ameliorated hyperuricemia-induced BA metabolic disorders.

#### 3.3.2. Association of BA Metabolism with UA Production via the Gut–Liver Axis

Building on the BA profile changes described above, we further explored the potential mechanism by which W6-12 modulates BA metabolism. In the present study, W6-12 intervention significantly reduced serum BA accumulation and increased fecal free BA levels, demonstrating marked changes in BA profiles consistent with an alleviation of BA enterohepatic circulation stasis. These changes were accompanied by a significant reduction in hepatic XOD activity, the key enzyme catalyzing endogenous UA synthesis.

BA metabolism has been reported to influence hepatic UA production through several pathways, including PPAR-α/XOD signaling and FXR/TGR5-dependent regulation of inflammation, and to shape gut microbiota composition in ways that affect UA homeostasis [35]. Among these, the intestinal FXR-FGF19 axis has been proposed as a regulator of BA enterohepatic circulation and hepatic lipid metabolism; suppression of this pathway has been associated with BA stasis and derepression of hepatic CYP7A1 in previous studies [36], and lower serum FGF19 levels have been reported in hyperuricemic patients [37]. A study using a cholesterol gallstone mouse model further reported that *L. reuteri* CGMCC 17942 activates intestinal FXR signaling and upregulates ileal FGF15, hepatic FGFR4, and SHP expression, thereby suppressing CYP7A1 [38]. These findings suggested that certain Lactobacillus strains may exert metabolic regulatory effects through BA-related signaling.

Consistent with this possibility, the present results showed that W6-12 reduced serum BA accumulation and increased fecal free BA levels, alongside a significant reduction in hepatic XOD activity. We therefore speculated that W6-12 may exert its hypouricemic effect through a combination of improved BA enterohepatic circulation and reduced hepatic XOD activity, possibly involving BA-related signaling pathways. However, FXR, FGF19/FGF15, and CYP7A1 were not directly measured in this study, and direct molecular evidence is required to confirm this proposed mechanism. This study added correlative support to the emerging concept that dysregulated BA metabolism contributes to gout and HUA progression [35], and provided new insights into probiotic-mediated HUA improvement via the BA-gut microbiota axis. Further molecular experiments are needed to validate this hypothesis.

### 3.4. Multi-Omics Association Analysis of the Gut Microbiota–Bile Acid–UA Metabolic Axis

To systematically dissect the intrinsic relationships among gut microbiota composition, BA profiles, UA metabolism, and hepatic and renal injury phenotypes, we performed Spearman correlation heatmap and Mantel test-based network analyses for multi-dimensional validation. Integrating pairwise microbiota–metabolite correlations and global metabolic differentiation across experimental groups, the potential mechanism underlying W6-12’s alleviation of HUA is proposed in this study, involving modulation of the BA-gut microbiota axis.

#### 3.4.1. Spearman Correlation Analysis of Gut Microbiota with Bile Acids and Hyperuricemia-Related Indicators

*Lactobacillus* abundance was significantly positively correlated with SUA concentration, hepatic XOD activity, serum IL-6 and IL-1β, renal MDA content, and renal urate transporters GLUT9 and URAT1. *Lactobacillus* abundance was also positively correlated with serum-accumulated primary and secondary BAs (including GCA, GCDCA, GLCA, and 7-Keto-DCA), while negatively correlated with multiple fecal excretory BAs. Several additional taxa, including *Lachnospiraceae_UCG-006*, *Coriobacteriaceae_UCG-002*, *Faecalibaculum*, *Anaerostipes*, and *Odoribacter*, also showed positive correlations with SUA. These correlations indicated that, in the present model, higher abundance of these taxa was associated with more severe UA overproduction, stronger inflammatory responses, and impaired enterohepatic circulation, consistent with the elevated *Lactobacillus* abundance observed in HUA rats and its reduction after W6-12 intervention (Section 3.2). Previous studies have reported that specific *Lactobacillus* strains, such as *L. plantarum* SQ001 and LLY-606, can lower SUA by inhibiting XOD activity and inflammatory mediator expression, or by blocking the TLR4/MyD88/NF-κB cascade [39,40]. Bile salt hydrolase (BSH) activity in *Lactobacillus* is recognized as a core functional trait modulating host BA homeostasis and supporting intestinal colonization [41], and *L. plantarum* LP104 has been shown to enhance fecal BA excretion [42]. However, the relationship between *Lactobacillus* abundance and HUA remained inconsistent, and this genus is known to exhibit substantial functional heterogeneity. The genus-level positive correlation observed here might therefore have reflected model- or strain-specific effects rather than a general property of the genus. The specific contribution of this genus to BA and UA metabolism in HUA remains to be determined. Collectively, these multi-omics correlations suggested that, in the present model, elevated abundance of *Lactobacillus* and related taxa was associated with hyperuricemic metabolic disorders, including excessive UA synthesis, systemic low-grade inflammation, and enterohepatic stasis.

In contrast, several taxa were significantly negatively correlated with SUA levels, hepatic XOD activity, and serum BA abundance, including *Ruminococcus*, *NK4A214_group*, *norank_f__[Eubacterium]coprostanoligenes_group*, *Turicibacter*, *Romboutsia*, *Corynebacterium*, *Lachnospira*, *Christensenellaceae_R-7_group*, *Monoglobus, norank_o__RF39*, and *Dubosiella*; these taxa were depleted in HUA rats but restored after W6-12 intervention. Among them, *Ruminococcus* and *norank_f_[Eubacterium]_coprostanoligenes_group* are known butyrate-producing bacteria, which are generally recognized as beneficial to intestinal barrier integrity and anti-inflammatory capacity. These correlations suggested that, in the present model, the loss of these taxa may be associated with HUA progression, and that their recovery following W6-12 intervention may contribute to the alleviation of hyperuricemic metabolic disorders. However, direct evidence linking these taxa to UA metabolism remains to be established.

With respect to secondary BA biotransformation, unclassified taxa within the *Lachnospiraceae* family were negatively correlated with fecal DCA content. Given the substantial inter- and intra-species functional heterogeneity within *Lachnospiraceae* [43], this correlation might have reflected the presence of non-7α-dehydroxylating members within this family. Whether these taxa contribute to DCA formation cannot be determined from genus-level 16S rRNA data, and metagenomic or functional-gene measurements of the bai gene cluster are required [44]. Notably, certain *NC2004 subgroups* within *Lachnospiraceae* have been reported to decrease under hyperuricemic conditions [45], indicating context-dependent, dual regulatory roles of this family in BA metabolism.

Correlations between the abundance of these taxa and BA-related indicators further suggested that, in the present model, the gut microbiota might have influenced host BA homeostasis through multiple pathways, including the intestinal FXR-FGF19 axis, which has been proposed to regulate BA enterohepatic circulation and hepatic lipid metabolism [46,47]. However, FXR activation and FGF19/FGF15 expression were not measured in the present study, and this possibility remains to be tested.

#### 3.4.2. Global Association Network Analysis Based on Mantel-Test

Mantel test-based global correlation analysis revealed distinct metabolic profiles among the three groups (Figure 5B). The HUA group exhibited comprehensive perturbations in UA metabolism, inflammatory responses, and BA homeostasis relative to the CON group. W6-12 intervention effectively rescued most hyperuricemia-induced metabolic abnormalities; nevertheless, evident metabolic differences persisted between the W6-12 and CON groups, indicating that W6-12 treatment achieved partial rather than complete restoration of physiological metabolic homeostasis. These results corroborated a previous multi-omics study, which demonstrated that HUA induced global dysregulation of gut microbiota, BA metabolism, and purine catabolism, and that probiotic intervention could only partially reverse these pathological metabolic shifts [35]. In summary, W6-12 supplementation reduced the abundance of *Lactobacillus* and related taxa associated with HUA severity, restored butyrate-producing and other negatively correlated taxa, increased fecal excretion of prototype primary BAs, and reduced serum BA accumulation. Based on the observed BA profile changes and the reduction in hepatic XOD activity, combined with existing literature on BA-related signaling, we hypothesized that W6-12 might exert its hypouricemic effect through BA-related pathways potentially involving the gut-liver axis. However, direct molecular evidence (e.g., FXR and FGF19 expression) is needed to confirm this proposed mechanism. The present multi-dimensional association analysis revealed robust crosstalk among gut microbiota, BA metabolism, UA anabolism, and renal injury phenotypes, providing correlative evidence for a potential hierarchical regulatory mechanism by which probiotic W6-12 might improve HUA via the BA-gut microbiota axis. Further molecular validation is still required to fully elucidate the underlying regulatory pathway.

### 3.5. Response Surface Optimization of Liquid Fermentation Medium

#### 3.5.1. Screening of Optimal Carbon and Nitrogen Sources and Their Optimal Concentrations

Carbon sources provide essential energy for the growth of lactic acid bacteria and directly determine the proliferation rate. Distinct strain-specific differences in carbon source utilization preferences and metabolic capacity were observed among different *L. reuteri* strains. *L. reuteri* CRL1100 exhibited better growth performance in sucrose-supplemented medium than in glucose-based medium and cannot utilize fructose as a sole carbon source [48]. Teixeira et al. [49] further clarified the underlying molecular mechanism: the presence of sucrose relieves the transcriptional repression of the sucrose metabolism regulatory gene *scrR* in *L. reuteri*, thereby enabling the efficient expression of functional genes responsible for sucrose degradation. *L. reuteri* DSM 17938 also showed significant discrepancies in specific growth rates when cultured with glucose, maltose, and sucrose, further verifying that carbon source type is a critical factor modulating the growth of this strain [50]. In this study, sucrose was identified as the optimal carbon source for *L. reuteri* W6-12 (Figure 6A), and the viable cell count presented a unimodal trend with the increase in sucrose concentration (Figure 6B). The maximum viable cell density was obtained at a sucrose concentration of 30 g/L, which was significantly higher than that at 25 g/L, and no further improvement in cell proliferation was observed when the sucrose concentration exceeded 30 g/L. Low concentrations of sucrose supply sufficient carbon skeletons and energy for strain growth, whereas excessive sucrose elevates medium osmotic pressure, forming a hyperosmotic microenvironment that inhibits cell proliferation and ultimately reduces viable cell counts. This growth pattern was consistent with the findings reported by Schwab et al. [51].

Considering production safety and raw material costs, five animal-free nitrogen sources, including peanut meal, cottonseed meal, soybean peptone, corn steep liquor, and yeast extract, were selected for screening. As illustrated in Figure 6C, yeast extract exhibited a significantly higher growth-promoting effects than other nitrogen sources. Rich in free amino acids, small molecular peptides, nucleotides, and B vitamins, yeast extract satisfies the complex nutritional requirements of *L. reuteri*, shortens the growth lag phase, and improves bacterial proliferation efficiency [52,53]. Previous studies have demonstrated that yeast extract supplementation increases the viable cell count of *L. reuteri* LR1 by nearly 37-fold, strongly confirming its prominent growth-promoting capacity [54]. Different *L. reuteri* strains exhibit distinct tolerance and optimal concentrations for yeast extract, with optimal doses of 44 g/L for DSM20016 [53] and 20 g/L for CG001 [55]. For strain W6-12, the maximum viable cell count of 1.24 × 10^9^ CFU/mL was achieved at a yeast extract concentration of 26 g/L with significant inter-group differences, and the viable cell count also increased first and then decreased with the elevation of yeast extract concentration (Figure 6D).

#### 3.5.2. Screening of Buffer Salt Concentrations

Continuous synthesis and secretion of organic acids such as lactic acid and acetic acid during lactic acid bacteria fermentation gradually decrease the medium pH, and acid stress severely inhibits cell proliferation and metabolic activity. Buffer salts can neutralize fermentation-derived organic acids, stabilize medium pH, maintain a favorable growth microenvironment, and improve bacterial biomass yield. The classical MRS medium adopts CH_3_COONa (5 g/L), C_6_H_14_N_2_O_7_ (2 g/L), and K_2_HPO_4_ (2 g/L) as core buffering components, among which CH_3_COONa and C_6_H_14_N_2_O_7_ can also effectively inhibit the contamination of Streptococcus and molds under low-pH conditions.

Based on the optimized carbon and nitrogen sources, the concentrations of three buffer salts (CH_3_COONa, K_2_HPO_4_, and C_6_H_14_N_2_O_7_) were further optimized for W6-12 cultivation. The optimal CH_3_COONa concentration for W6-12 was determined as 7.5 g/L, which was higher than the standard MRS medium dosage (5 g/L), presumably attributed to the high acid-producing characteristics and strong osmotic tolerance of this strain. The optimal concentrations of K_2_HPO_4_ (1 g/L) and C_6_H_14_N_2_O_7_ (3 g/L) were close to the standard MRS formulation. Heidarrezaei et al. [53] optimized the medium for *L. reuteri* DSM20016 and obtained optimal concentrations of 2.6 g/L for CH_3_COONa, 0.2 g/L for C_6_H_14_N_2_O_7_, and 0.2 g/L for K_2_HPO_4_, with a much lower CH_3_COONa dosage than the MRS standard, indicating that the buffer salt requirements of *L. reuteri* are highly strain-specific. The synergistic effect of the three buffer salts efficiently stabilized fermentation pH and sustained the continuous proliferation of W6-12 during high-density cultivation.

#### 3.5.3. Screening of Trace Elements and Tween-80 Concentrations

Trace elements, though supplied in small amounts, are critically involved in regulating the growth, metabolism, and metabolite production of lactic acid bacteria. In this study, the optimal concentrations of MnSO_4_ and MgSO_4_ for W6-12 cultivation were explored. As shown in Figure 6H,I, the optimal concentrations were 0.075 g/L for MnSO_4_ and 0.1 g/L for MgSO_4_, which yielded significantly higher viable cell counts than other concentration gradients. Mn^2+^ and Mg^2+^ act as essential cofactors for key intracellular enzymes of lactic acid bacteria. Mn^2+^ activates superoxide dismutase, kinases, and decarboxylases, alleviating intracellular oxidative damage and maintaining normal glycolytic flux. Mg^2+^ functions as an ATPase activator in energy metabolism and stabilizes ribosomal structure, thereby ensuring normal bacterial protein synthesis. Chen et al. [55] conducted Plackett–Burman experiments on *L. reuteri* CG001 and verified that MnSO_4_ was a core factor affecting strain growth, with an optimized concentration of 0.23 g/L determined via response surface methodology, which was higher than the 0.075 g/L obtained in this study, reflecting strain-specific differences in manganese demand. Heidarrezaei et al. [53] optimized the medium for *L. reuteri* DSM20016T and reported MgSO_4_ and MnSO_4_ concentrations of 0.2 g/L and 0.05 g/L, respectively; the MgSO_4_ concentration was higher than that in the present study (0.1 g/L), while the MnSO_4_ concentration was basically consistent. Most previous studies on lactic acid bacteria medium optimization have adopted MgSO_4_ heptahydrate and MnSO_4_ monohydrate at concentrations ranging from 0.01 to 0.25 g/L, and the optimized concentrations in this study fall within this universal range. The numerical differences from previous reports can be explained by strain specificity, variations in basal medium composition, and different evaluation indicators (viable cell count vs. cell dry weight).

Tween-80 is a widely used non-ionic surfactant in lactic acid bacteria culture media. Beyond its emulsifying function, it exerts multiple regulatory effects on bacterial growth, metabolism, and stress resistance. As demonstrated in Figure 6J, the optimal Tween-80 concentration for W6-12 proliferation was 1 g/L. Mechanistically, Tween-80 releases exogenous unsaturated fatty acids represented by oleic acid, which are absorbed by bacterial cells and integrated into the cell membrane phospholipid bilayer. This process increases the unsaturation degree of membrane fatty acids, enhances cell membrane fluidity, and improves bacterial tolerance to acid stress, osmotic pressure, and freeze-drying damage. Qian et al. [56] reported that supplementation of 1.8 g/L Tween-80 in MRS liquid medium increased the membrane fatty acid unsaturation rate of *Levilactobacillus brevis* 173-1-2 from 64.14% to 80.31% and raised the lyophilization survival rate from 41.87% to 60.72%; similarly, the unsaturation rate and lyophilization survival rate of *Lacticaseibacillus rhamnosus* FJND increased from 54.01% to 74.72% and 45.22% to 59.63%, respectively. Consistent with our results, Chen et al. [55] and Heidarrezaei et al. [53] both adopted 1 g/L Tween-80 in the culture media for *L. reuteri* CG001 and DSM20016. Notably, the universal applicable dosage range of Tween-80 in bacterial media is 0.9-1.0 g/L, and excessive concentrations cause surfactant cytotoxicity and inhibit bacterial growth. The optimal concentration determined in this study conformed to the industry universal formulation, exhibiting good applicability.

#### 3.5.4. Plackett-Burman Screening

A Plackett-Burman design was performed to further screen the most significant factors among the eight candidates. Based on the ANOVA, Pareto chart, and normal probability plot, sucrose, yeast extract, and CH_3_COONa were identified as the most significant factors affecting viable cell count. These three factors were therefore selected for subsequent Box-Behnken optimization. The results are provided in Supplementary Tables S1 and S2 and Supplementary Figure S3.

#### 3.5.5. Response Surface Optimization

Using a three-factor, three-level Box-Behnken design, three critical medium components, namely sucrose (A), yeast extract (B), and CH_3_COONa (C), were selected for optimization. The 17 experimental runs and their results are provided in Table 1. Design-Expert 13.0 software was used to perform multiple regression fitting on the experimental data, and the quadratic polynomial regression equation for viable cell count (Y) was established as follows: Y = 1.96 + 0.1162A + 0.5000B − 0.0637C − 0.0675AB + 0.0900AC + 0.0125BC − 0.2975A^2^ − 0.4500B^2^ − 0.3225C^2^. The regression model was found to be extremely significant (*p* < 0.0001) according to ANOVA results (Supplementary Table S3), confirming adequate fit of the established model and reliable predictive performance for medium formulation optimization, with an R^2^ of 0.9836, an adjusted R^2^ of 0.9624, a predicted R^2^ of 0.8470, a coefficient of variation of 4.82%, and an adequate precision of 18.2306. The lack-of-fit was not significant (*p* > 0.05). Residual diagnostic plots are provided in Supplementary Figure S4. Significance analysis of regression coefficients showed that all linear terms (A, B, C) and quadratic terms (A^2^, B^2^, C^2^) exerted extremely significant effects on viable cell count (*p* < 0.01), and the interaction term AC showed a significant effect (*p* < 0.05), suggesting an obvious synergistic interaction between sucrose and CH_3_COONa.

The interactive relationships among the three factors were visualized by the three-dimensional response surface and contour plots shown in Figure 7. With the increase in sucrose and yeast extract concentrations, the viable cell count increased first and then decreased, which was consistent with the variation trend observed in single-factor experiments. Model prediction showed that the optimal medium formulation consisted of 30.90 g/L sucrose, 26.17 g/L yeast extract, and 7.35 g/L CH_3_COONa, with a theoretical maximum viable cell count of 1.97 × 10^9^ CFU/mL. Considering the convenience of practical experimental operation, the formulation was appropriately revised to 31.00 g/L sucrose, 26.00 g/L yeast extract, and 7.50 g/L CH_3_COONa. Three parallel validation experiments were carried out using the revised formulation, and the average measured viable cell count reached 2.03 × 10^9^ CFU/mL. No significant difference was observed between the measured and predicted values (*p* > 0.05), which confirmed that the regression model was reliable for prediction and the optimized fermentation medium formulation was feasible and effective.

### 3.6. Response Surface Optimization of Lyophilization Cryoprotectants

#### 3.6.1. Screening of Cryoprotectant Types and Concentrations

The effects of 17 candidate cryoprotectants at 10% concentration on the lyophilization survival rate of *L. reuteri* W6-12 were evaluated. As shown in Figure 8A, different protectants exhibited significantly different protective effects against freeze-drying damage, indicating that cryoprotectant type is a key factor determining the lyophilization survival rate of the strain. Among the tested protectants, three single protectants—L-sodium glutamate, skim milk powder and trehalose—showed significantly higher lyophilization survival rates than the other 14 protectants, with corresponding survival rates of 80.85%, 81.88% and 78.96%, respectively. These three were selected as candidate protectants for further optimization.

Single-factor experiments were conducted for these three protectants. Viable cell counts before and after lyophilization were measured, and survival rates were calculated. As shown in Figure 8B–D, the lyophilization survival rate of W6-12 first increased and then decreased with increasing protectant concentration for all three protectants. The optimal concentrations for individual protectants were 8% L-sodium glutamate, 10% trehalose, and 12% skim milk powder.

#### 3.6.2. Determination of Optimal Protectant Formulation by Response Surface Methodology

The 17 experimental runs and their results are provided in Table 2. Through ANOVA and quadratic polynomial regression, the following multiple quadratic regression equation for L-sodium glutamate (A), trehalose (B), skim milk powder (C), and survival rate (Y) was obtained: Y = 88.14 + 5.26A − 3.86B + 3.42C − 0.5250AB − 3.86AC + 3.33BC − 15.21A^2^ − 15.04B^2^ − 8.02C^2^. This equation accurately represented the quantitative relationship between the three protectant concentrations and the lyophilization survival rate of W6-12. As indicated by ANOVA results (Supplementary Table S4), the regression model reached a high significance level (*p* < 0.0001), indicating good predictive quality of the model for cryoprotectant formulation optimization, with an R^2^ of 0.9828, an adjusted R^2^ of 0.9608, a predicted R^2^ of 0.8471, a coefficient of variation of 3.88%, and an adequate precision of 18.6008. The lack-of-fit value was non-significant (*p* > 0.05). Residual diagnostic plots are provided in Supplementary Figure S5. The quadratic terms (A^2^, B^2^, C^2^) and the interaction term AC all had significant effects on survival rate, indicating that the model could be used to optimize the cryoprotectant formulation.

To visualize the interaction effects among the three factors, three-dimensional response surface and contour plots were generated using Design-Expert 13.0, as shown in Figure 9. Contour plots approaching elliptical shapes indicated more significant interactions between factors. All response surfaces exhibited a bell-shaped pattern, suggesting that an optimal combination of the three protectants existed within the tested concentration range to achieve maximum lyophilization survival. Model optimization predicted the optimal cryoprotectant formulation as 8.62% L-sodium glutamate, 9.54% trehalose, and 12.61% skim milk powder, with a predicted survival rate of 89.03%. Considering experimental convenience, the formulation was adjusted to 8.00% L-sodium glutamate, 10.00% trehalose, and 12.00% skim milk powder. Multiple repeated validation experiments yielded an average survival rate of 89.47%, showing no significant deviation from the predicted value and confirming the reliability of the response surface model and the practical applicability of the optimized composite cryoprotectant formulation.

### 3.7. In Vivo Efficacy Validation of Lyophilized Bacterial Powder

To verify whether the W6-12 lyophilized powder prepared through optimized fermentation and lyophilization processes retained the hypouricemic efficacy of the live bacterial suspension, different doses of the powder were administered to HUA rats by gavage. W6-12 lyophilized powder produced a dose-dependent reduction in SUA levels in HUA rats (Figure 10A). Serum CRE and BUN were significantly reduced by low, medium, and high doses of W6-12 powder relative to the HUA model group (Figure 10B,C), suggesting attenuation of hyperuricemia-induced renal damage. HE-stained kidney sections (Figure 10D) from the HUA model group revealed widespread vacuolar degeneration of tubular epithelial cells, tubular dilation, and substantial inflammatory cell infiltration, indicating severe renal pathological injury. After high-dose W6-12 powder intervention, the structural integrity of renal tubules was significantly restored, and renal inflammatory lesions and cellular degeneration were markedly alleviated. Collectively, these results confirmed that the W6-12 lyophilized powder prepared via optimized fermentation and cryoprotectant processes retained remarkable urate-lowering and kidney-protective properties in vivo, and these physiological effects were enhanced with increasing powder dosage, displaying a clear dose-dependent pattern.

## 4. Conclusions

This study evaluated the hypouricemic effect of *L. reuteri* W6-12 and optimized its production process. W6-12 significantly reduced SUA levels and alleviated renal injury in HUA rats, primarily through inhibition of hepatic XOD activity, downregulation of renal GLUT9 and URAT1, and attenuation of renal oxidative stress and inflammation. 16S rRNA sequencing and untargeted metabolomics further revealed that W6-12 reversed gut microbiota dysbiosis, reducing *Lactobacillus* and related taxa associated with HUA severity while restoring butyrate-producing taxa, alleviated bile acid enterohepatic circulation stasis, and modulated serum and fecal metabolite profiles. Multi-omics correlation analysis suggested that these effects may involve the gut microbiota–bile acid axis, possibly through FXR–FGF19 signaling; however, FXR, FGF19/FGF15, and CYP7A1 were not directly measured, and direct molecular evidence is required to confirm this proposed mechanism. In parallel, an optimal fermentation medium and an optimal composite cryoprotectant were established, achieving a viable cell count of 2.03 × 10^9^ CFU/mL and a lyophilization survival rate of 89.47%, respectively, and the resulting lyophilized powder exhibited dose-dependent hypouricemic activity in HUA rats. Nevertheless, this study was conducted at laboratory scale and was limited to a chemically induced rat model. The efficacy, safety, optimal human dosage, and clinical relevance of W6-12 remain to be established, and larger animal sample sizes are needed to further validate these findings, and further studies are required to evaluate scale-up performance, batch-to-batch reproducibility, production yield, process economics, storage stability, and long-term viability before industrial application can be considered. Additional toxicity and safety studies, independent validation in other models, and human clinical trials are needed before the translational potential of W6-12 can be fully assessed.

## Figures and Tables

**Figure 1:**
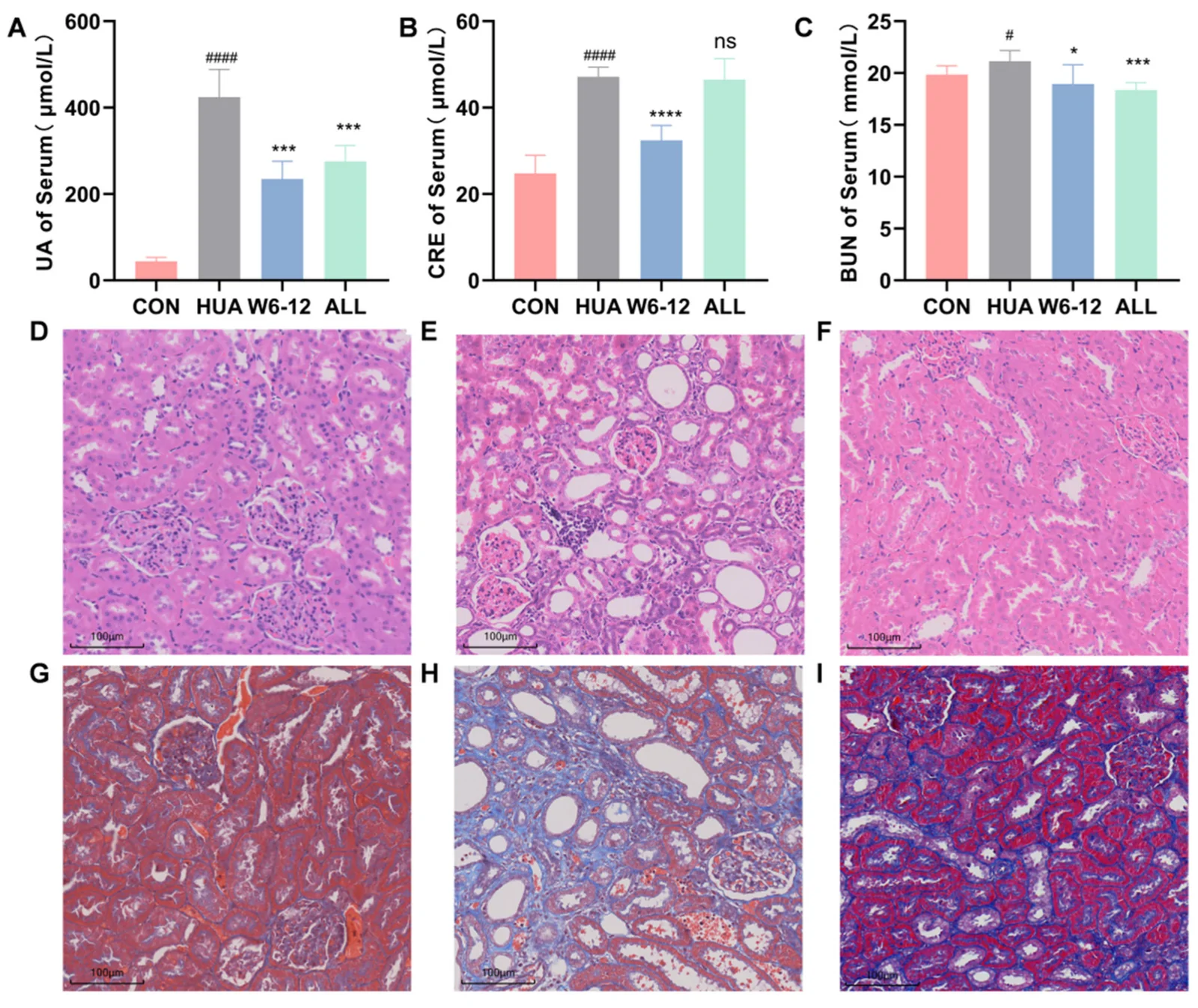
Serum UA levels and renal function in the CON, HUA, and W6-12 groups. (**A**) Serum UA. (**B**) Serum CRE. (**C**) Serum BUN. (**D**–**F**) Representative HE staining images of kidney sections, CON, HUA, W6-12 from left to right (scale bar: 100 μm). (**G**–**I**) Representative Masson’s trichrome staining images of kidney sections, CON, HUA, W6-12 from left to right (scale bar: 100 μm). Data are mean ± SD (*n* = 6). ^#^ *p* < 0.05, ^####^ *p* < 0.0001; * *p* < 0.05, *** *p* < 0.001, **** *p* < 0.0001; ns, not significant.

**Figure 2:**
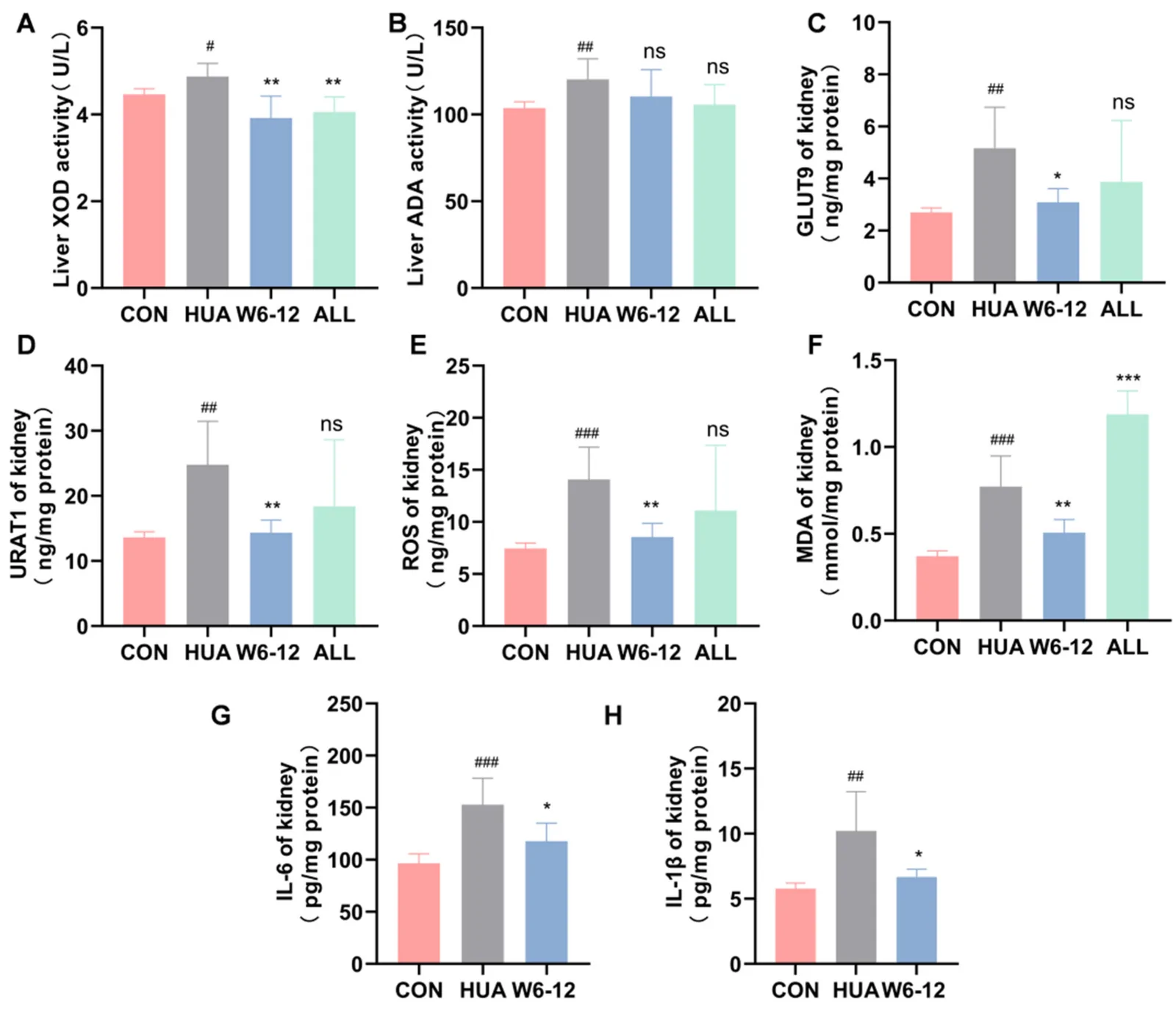
Hepatic purine metabolic enzyme activities, renal urate transporters, and renal oxidative stress and inflammatory markers in the CON, HUA, and W6-12 groups. (**A**) XOD activity. (**B**) ADA activity. (**C**) GLUT9. (**D**) URAT1. (**E**) ROS. (**F**) MDA. (**G**) IL-6. (**H**) IL-1β. Data are mean ± SD (*n* = 6). ^#^ *p* < 0.05, ^##^ *p* < 0.01, ^###^ *p* < 0.001; * *p* < 0.05, ** *p* < 0.01, *** *p* < 0.001; ns, not significant.

**Figure 3:**
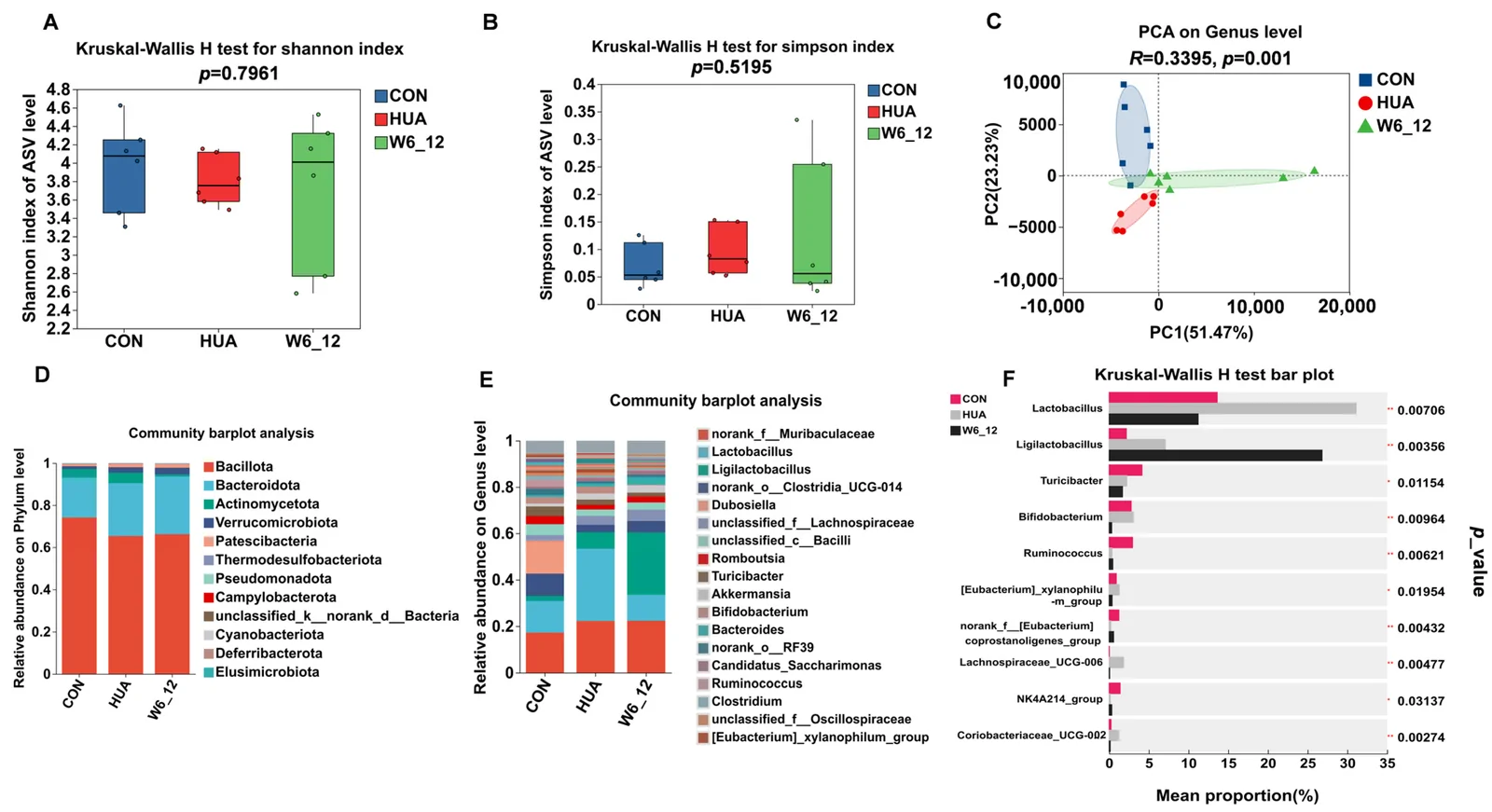
Gut microbiota diversity and composition in the CON, HUA, and W6-12 groups. (**A**,**B**) Shannon and Simpson index of the gut microbiota in each group. (**C**) PCA score plot of the gut microbiota based on beta diversity, where each point represents one sample. (**D**,**E**) Relative abundance of the dominant bacterial phylum and genus in each group. (**F**) Multi-group comparison of differentially abundant genera among the three groups. Data are mean ± SD (*n* = 6), * *p* < 0.05, ** *p* < 0.01.

**Figure 4:**
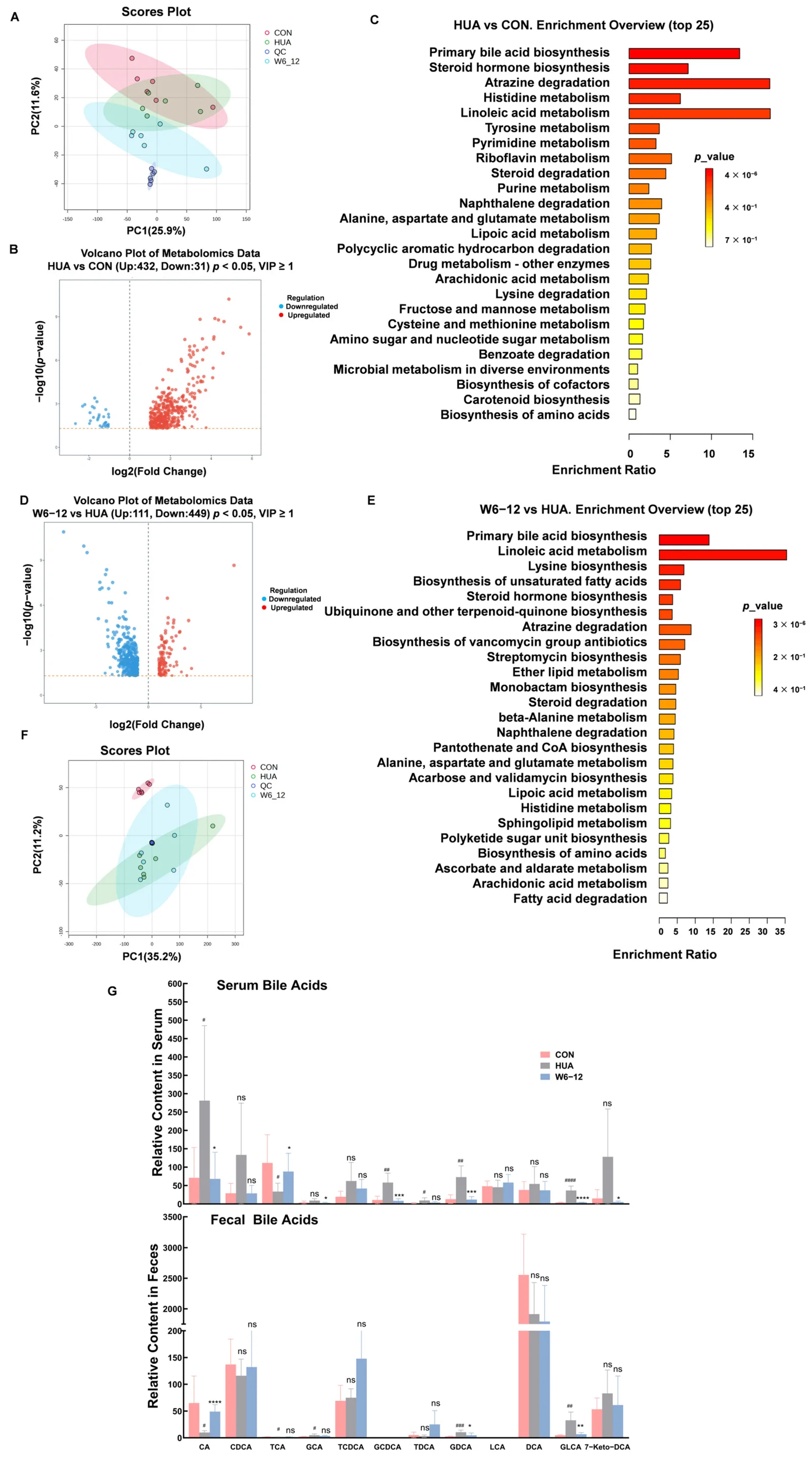
Serum and fecal metabolite profiles in the CON, HUA, and W6-12 groups. (**A**) PCA score plot of the serum metabolome, where each point represents one sample. (**B**) Volcano plot of serum metabolites between the HUA and CON groups. (**C**) KEGG pathway enrichment analysis of serum metabolites between the HUA and CON groups. (**D**) Volcano plot of serum metabolites between the W6-12 and HUA groups. (**E**) KEGG pathway enrichment analysis of serum metabolites between the W6-12 and HUA groups. (**F**) PCA score plot of the fecal metabolome, where each point represents one sample. (**G**) Heatmap of serum and fecal BAs profiles, ^#^ *p* < 0.05, ^##^ *p* < 0.01, ^###^ *p* < 0.001, ^####^ *p* < 0.0001; * *p* < 0.05, ** *p* < 0.01, *** *p* < 0.001, **** *p* < 0.0001; ns, not significant. Differential metabolites were selected based on VIP > 1, |log_2_FC| ≥ 1, and unadjusted *p* < 0.05. In (**B**,**D**), the horizontal dashed line indicates log_2_FC = 0, and the vertical dashed line indicates VIP = 1.

**Figure 5:**
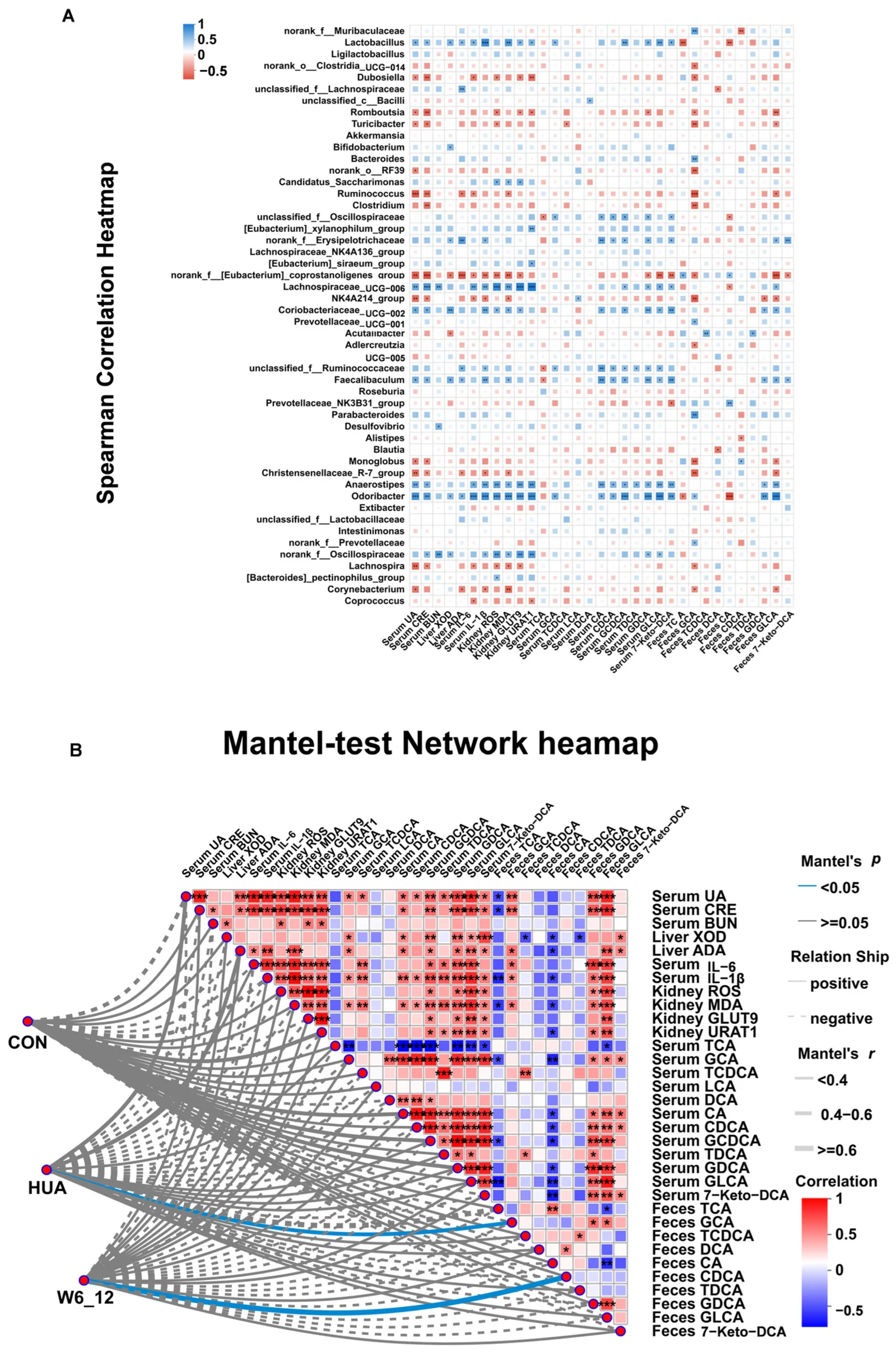
Multi-omics association analysis of the gut microbiota-bile acid-uric acid metabolic axis. (**A**) Spearman correlation heatmap between gut microbial genera and uric acid-related indicators, inflammatory cytokines, oxidative stress markers, renal transporters, and serum/fecal BAs. Correlation color code: red (positive), blue (negative). * *p* < 0.05, ** *p* < 0.01. *** *p* < 0.001. (**B**) Mantel test-based global association network showing the correlations among gut microbiota, BA metabolism, UA metabolism, and renal injury phenotypes. Line width and color indicate correlation strength and significance level, respectively. * *p* < 0.05, ** *p* < 0.01. *** *p* < 0.001.

**Figure 6:**
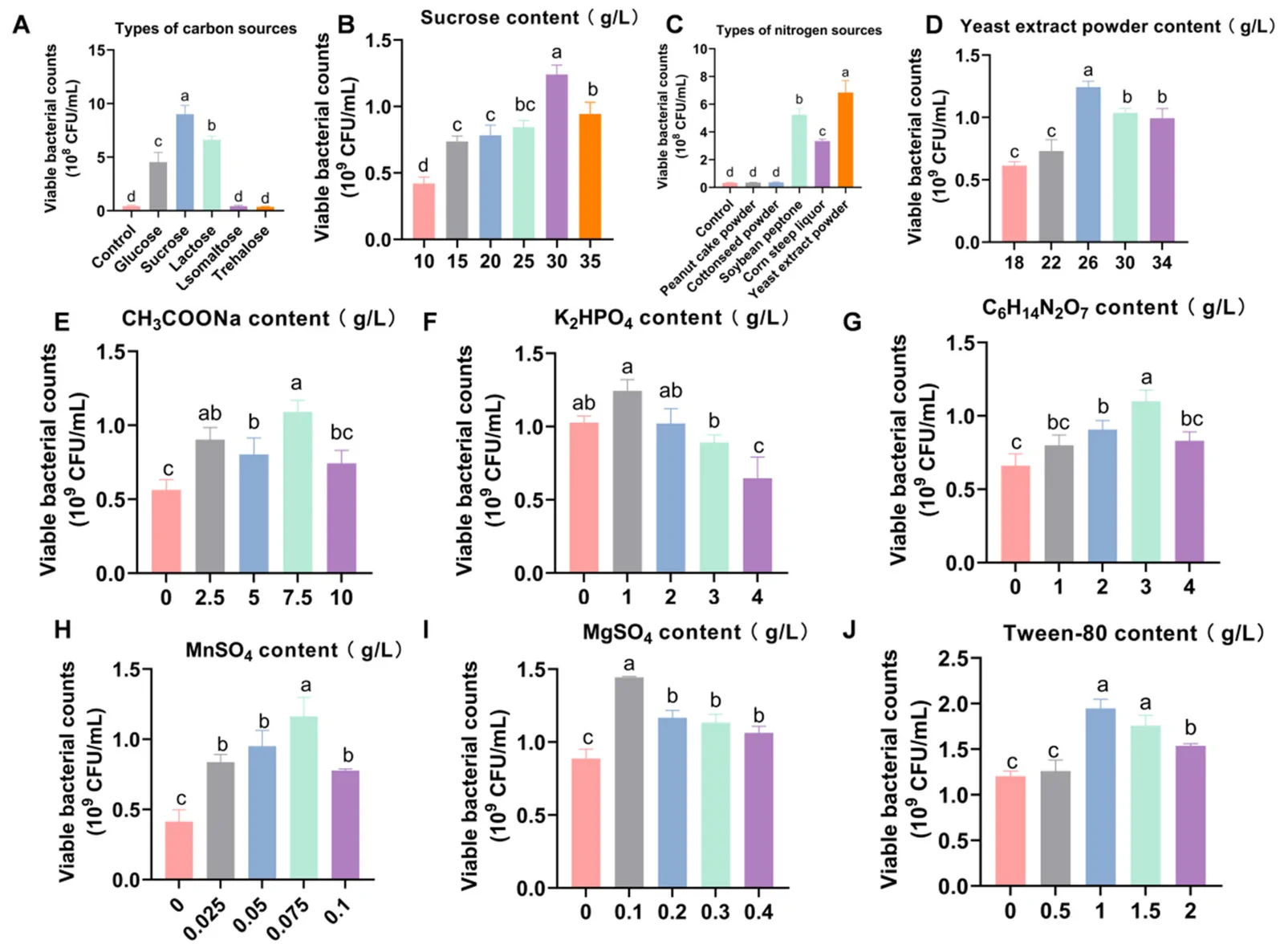
Single-factor optimization of fermentation medium components for *L. reuteri* W6-12. (**A**) Viable cell count with different carbon sources. (**B**) Viable cell count at varying sucrose concentrations. (**C**) Viable cell count with different nitrogen sources. (**D**) Viable cell count at varying yeast extract concentrations. (**E**–**G**) Effects of buffer salts (CH_3_COONa, K_2_HPO_4_, and C_6_H_14_N_2_O_7_) on viable cell count. (**H**–**I**) Effects of trace elements (MnSO_4_ and MgSO_4_) on viable cell count. (**J**) Viable cell count at varying Tween-80 concentrations. Data are mean ± SD (*n* = 3). Different letters (a–d) denote significant differences among groups (one-way ANOVA with Tukey’s post hoc test, *p* < 0.05).

**Figure 7:**
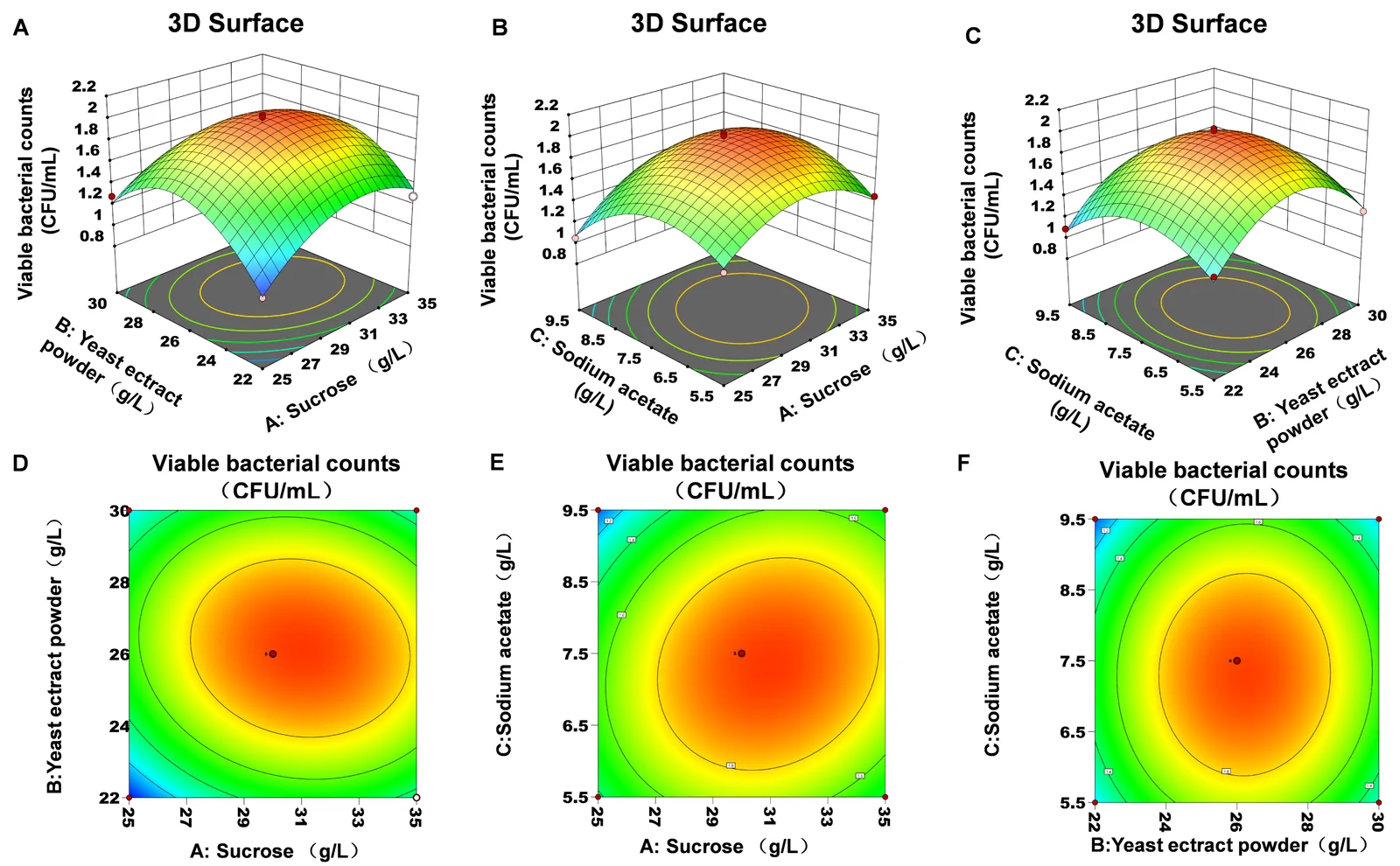
Response surface and contour plots of the interactive effects among fermentation medium components. (**A**–**C**) Contour plots of viable cell count as a function of sucrose vs. yeast extract (**A**), sucrose vs. CH_3_COONa (**B**), and yeast extract vs. CH_3_COONa (**C**). (**D**–**F**) Corresponding three-dimensional response surface plots for sucrose and yeast extract (**D**), sucrose and CH_3_COONa (**E**), and yeast extract and CH_3_COONa (**F**). Elliptical contour shapes indicate significant interactions between the two factors. The optimal regions are highlighted in red.

**Figure 8:**
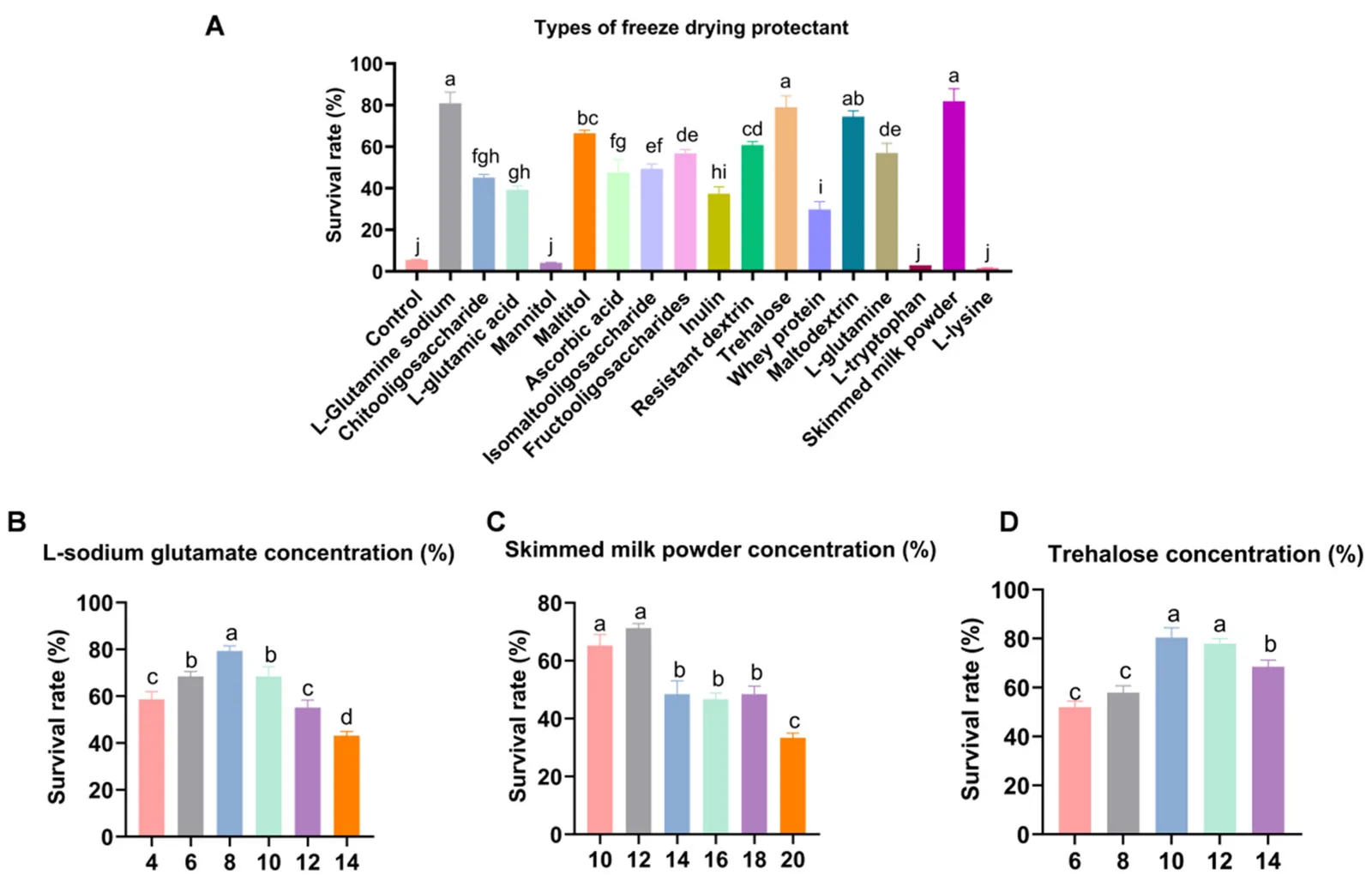
Single-factor optimization of cryoprotectants for *L. reuteri* W6-12 lyophilization. (**A**) Lyophilization survival rates of 17 candidate cryoprotectants at 10% (*w*/*v*) concentration. (**B**–**D**) Effects of L-sodium glutamate, skim milk powder and trehalose concentrations on lyophilization survival rate. Data are mean ± SD (*n* = 3). Groups with different letters (a–j) differ significantly (Tukey’s test after one-way ANOVA, *p* < 0.05).

**Figure 9:**
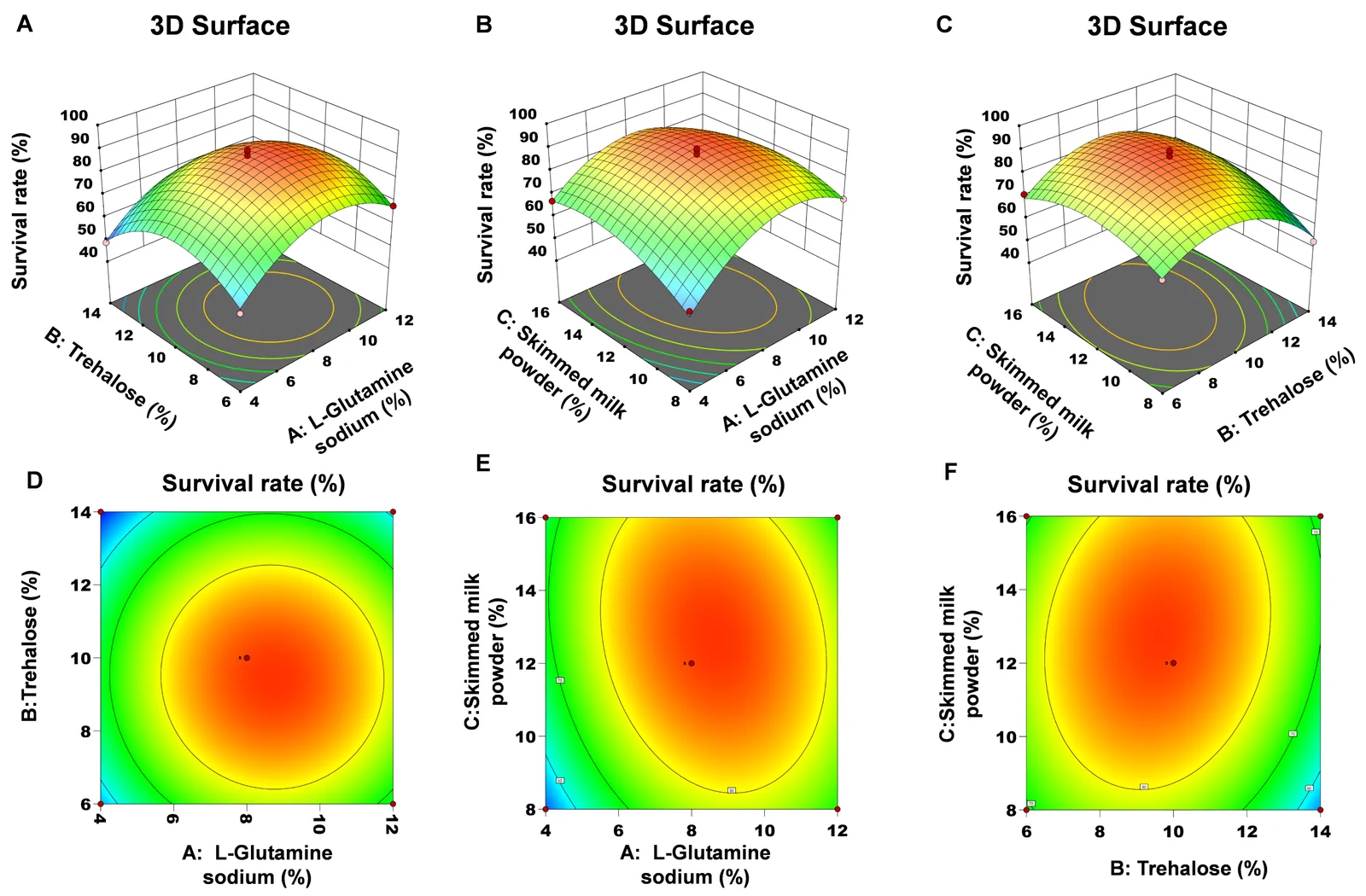
Response surface and contour plots of the interactive effects among cryoprotectant formulation components. (**A**–**C**) Contour plots of lyophilization survival rate as a function of L-sodium glutamate vs. trehalose (**A**), L-sodium glutamate vs. skim milk powder (**B**), and trehalose vs. skim milk powder (**C**). (**D**–**F**) Three-dimensional response surface plots for the corresponding factor combinations: L-sodium glutamate and trehalose (**D**), L-sodium glutamate and skim milk powder (**E**), and trehalose and skim milk powder (**F**). Elliptical contours indicate significant interactions between factors. Optimal regions are highlighted in red.

**Figure 10:**
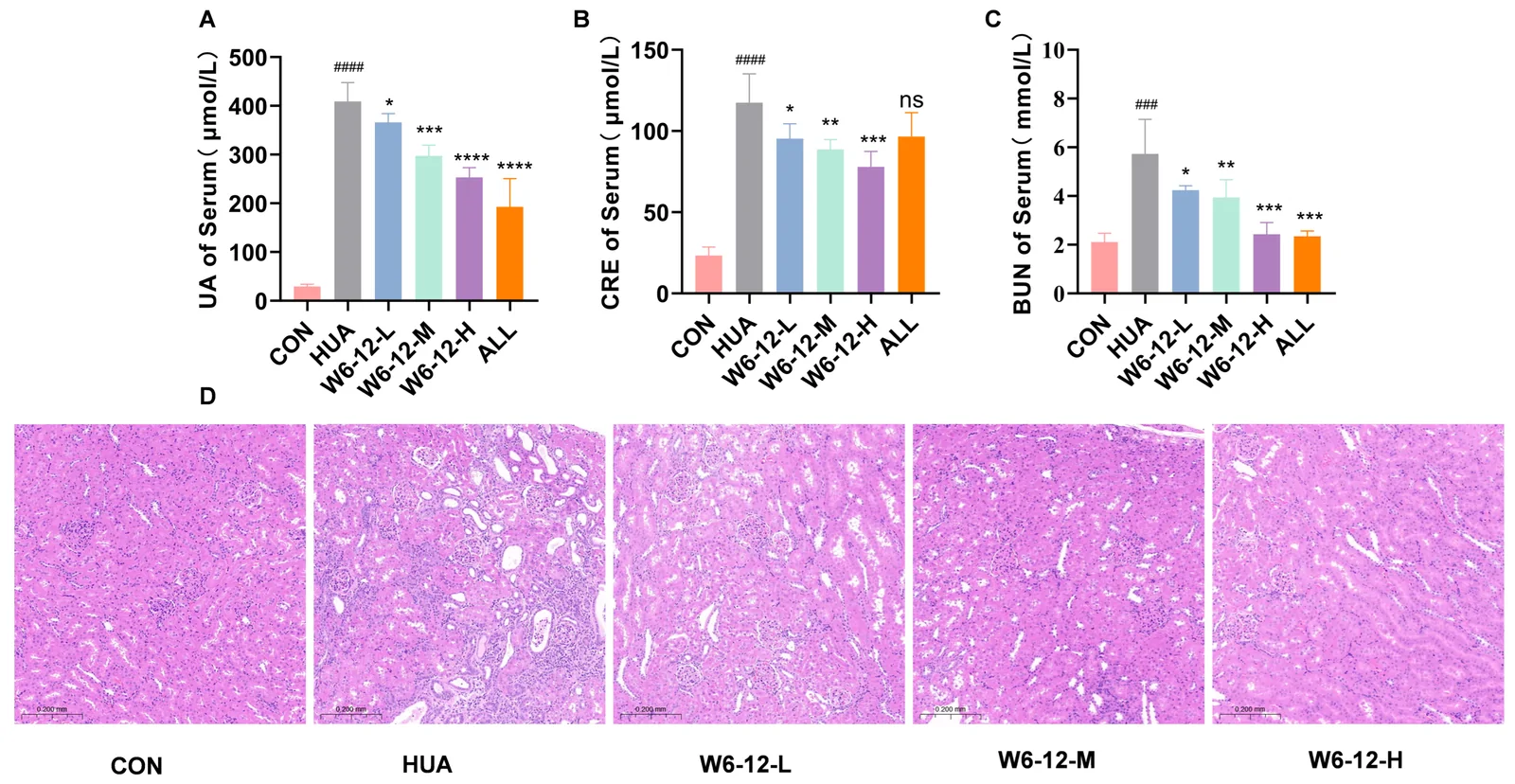
Dose-dependent effects of W6-12 lyophilized powder on Serum UA levels and renal function in HUA rats. (**A**) Serum UA. (**B**) Serum CRE. (**C**) Serum BUN. (**D**) HE staining of kidney sections (scale bar: 200 μm). Data are mean ± SD (*n* = 6). ^###^ *p* < 0.001, ^####^ *p* < 0.0001; * *p* < 0.05, ** *p* < 0.01, *** *p* < 0.001, **** *p* < 0.0001; ns, not significant.

**Table 1:** Box-Behnken design and results for the optimization of fermentation medium components for *L. reuteri* W6-12.

Group/Factor	Content (g/L)			Viable Bacterial Count (10^9^ CFU/mL)
	Sucrose	Yeast Extract Powder	CH_3_COONa	
1	25	30	7.5	1.28
2	35	26	5.5	1.45
3	30	26	7.5	2.03
4	35	22	7.5	1.28
5	30	30	5.5	1.32
6	30	30	9.5	1.14
7	30	22	5.5	1.26
8	25	26	9.5	1.05
9	30	22	9.5	1.09
10	30	26	7.5	2.01
11	30	26	7.5	1.87
12	25	26	5.5	1.34
13	35	26	9.5	1.52
14	30	26	7.5	1.94
15	35	30	7.5	1.32
16	25	22	7.5	0.97
17	30	26	7.5	1.95

Data are mean (*n* = 3).

**Table 2:** Box-Behnken design and results for the optimization of cryoprotectant formulation for *L. reuteri* W6-12 lyophilization.

Group/Factor	Concentration (%)			Survival Rate (%)
	L-Sodium Glutamate	Trehalose	Skim Milk Powder	
1	6	4	5	70.53
2	4	8	7	68.42
3	6	8	5	67.37
4	2	8	5	67.02
5	4	4	7	52.63
6	6	6	7	60.70
7	4	6	5	84.91
8	6	6	3	68.07
9	4	8	3	70.88
10	4	4	3	68.42
11	4	6	5	90.18
12	2	6	7	48.77
13	2	6	3	54.04
14	4	6	5	90.88
15	2	4	5	54.74
16	4	6	5	86.32
17	4	6	5	88.42

Data are mean (*n* = 3).

## Data Availability

The original contributions presented in this study are included in the article and Supplementary Material. Further inquiries can be directed to the corresponding authors.

## Supplementary Materials

The following supporting information can be downloaded at: https://www.mdpi.com/article/10.3390/foods15193451/s1, Figure S1: Additional analyses of gut microbiota diversity and differential taxa in the CON, HUA, and W6-12 groups. (A–C) Chao1, ACE, and Sobs indices of the gut microbiota in each group. (D) Venn diagram showing the shared and unique microbial genera among the three groups. (E) LEfSe analysis (LDA > 2, *p* < 0.05) showing differentially enriched taxa among the three groups. (F) Circos plot illustrating the distribution of gut microbial genera among the three groups. Data are mean ± SD (*n* = 6); Figure S2: Fecal metabolite profiles in the CON, HUA, and W6-12 groups. (A) Volcano plot of fecal metabolites between the HUA and CON groups. (B) KEGG pathway enrichment analysis of fecal metabolites between the HUA and CON groups. (C) Volcano plot of fecal metabolites between the W6-12 and HUA groups. (D) KEGG pathway enrichment analysis of fecal metabolites between the W6-12 and HUA groups; Figure S3: Plackett-Burman analysis of the factors affecting viable cell count of *L. reuteri* W6-12. (A) Normal plot. (B) Pareto chart; Figure S4: Residual diagnostic plots for the Box-Behnken model of fermentation medium optimization. (A) Normal probability plot of residuals. (B) Plot of predicted vs. actual values. (C) Plot of residuals vs. predicted values; Figure S5: Residual diagnostic plots for the Box-Behnken model of cryoprotectant formulation optimization. (A) Normal probability plot of residuals. (B) Plot of predicted vs. actual values. (C) Plot of residuals vs. predicted values; Table S1: Plackett-Burman experimental design and results for screening significant factors affecting viable cell count of *L. reuteri* W6-12; Table S2: ANOVA for the Plackett-Burman design of fermentation medium optimization; Table S3: ANOVA results for the response surface quadratic model of fermentation medium optimization; Table S4: ANOVA results for the response surface quadratic model of cryoprotectant formulation optimization.
