# Supplementary material for: From Efficacy to Process: Uric-Acid-Lowering Effect of Limosilactobacillus reuteri W6-12 and Its Production Process Optimization
Source: Foods. 2026 Sep 27;15(19):3451. doi: 10.3390/foods15193451 (PMC13664027; doi:10.3390/foods15193451)
Supplement: Supplementary file 1 [file foods-15-03451-s001.zip › foods-4574168-supplementary.pdf]

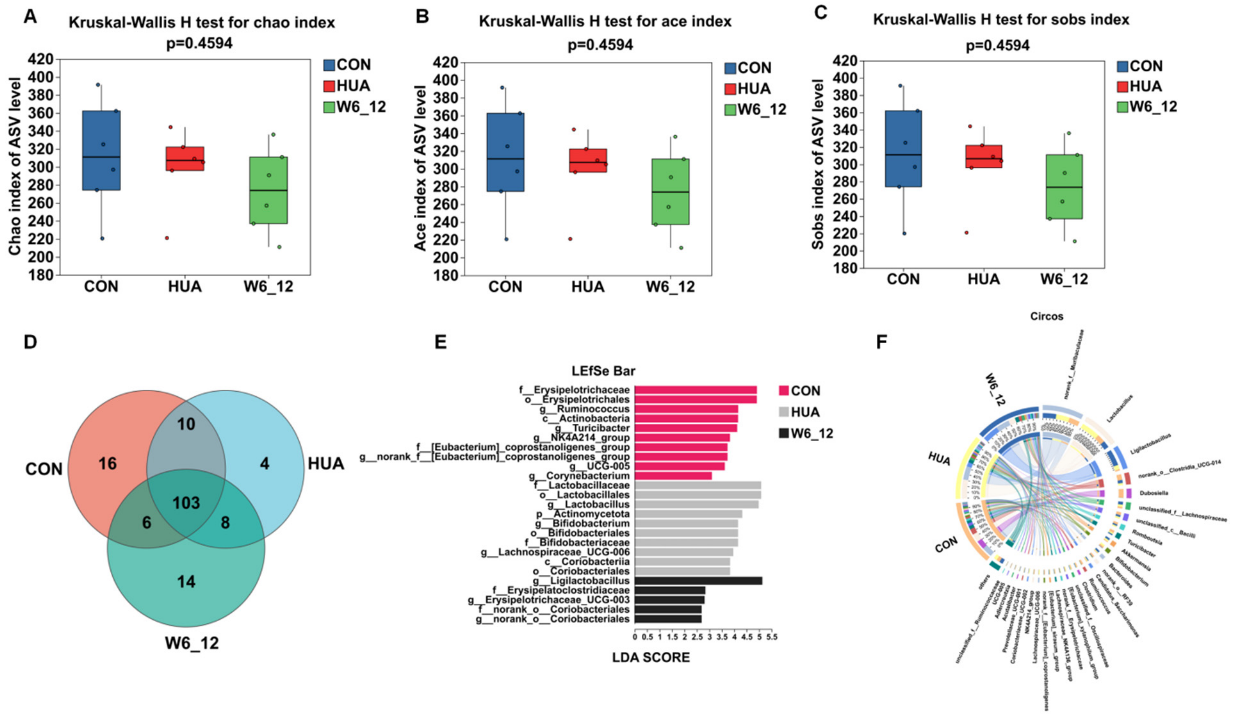

Supplementary Figure S1. Additional analyses of gut microbiota diversity and differential taxa in the CON, HUA, and W6-12 groups. (A-C) Chao1, ACE, and Sobs indices of the gut microbiota in each group. (D) Venn diagram showing the shared and unique microbial genera among the three groups. (E) LEfSe analysis (LDA > 2, P < 0.05) showing differentially enriched taxa among the three groups. (F) Circos plot illustrating the distribution of gut microbial genera among the three groups. Data are mean ± SD (n = 6).

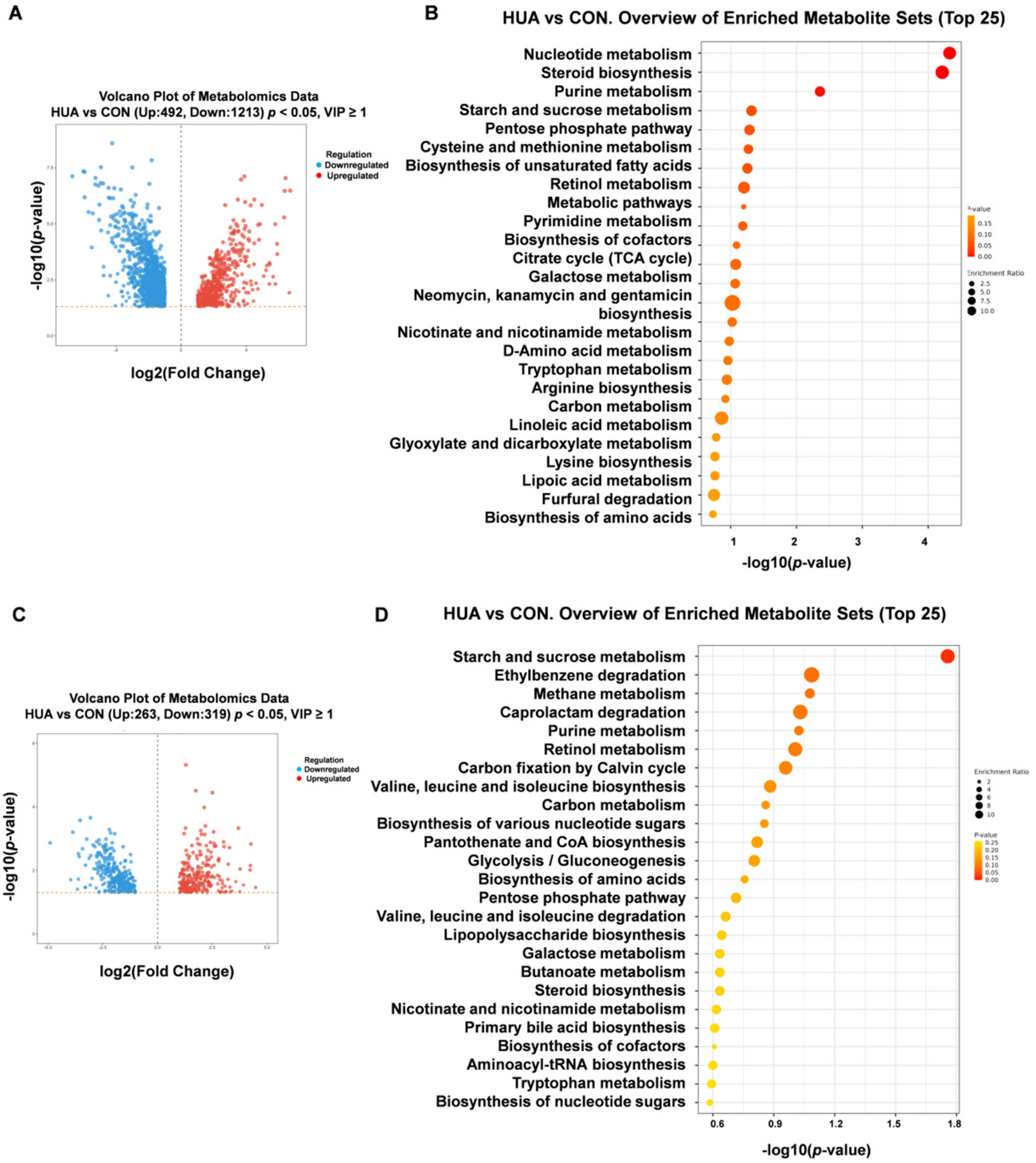

Supplementary Figure S2. Fecal metabolite profiles in the CON, HUA, and W6-12 groups. (A) Volcano plot of fecal metabolites between the HUA and CON groups. (B) KEGG pathway enrichment analysis of fecal metabolites between the HUA and CON groups. (C) Volcano plot of fecal metabolites between the W6-12 and HUA groups. (D) KEGG pathway enrichment analysis of fecal metabolites between the W6-12 and HUA groups.

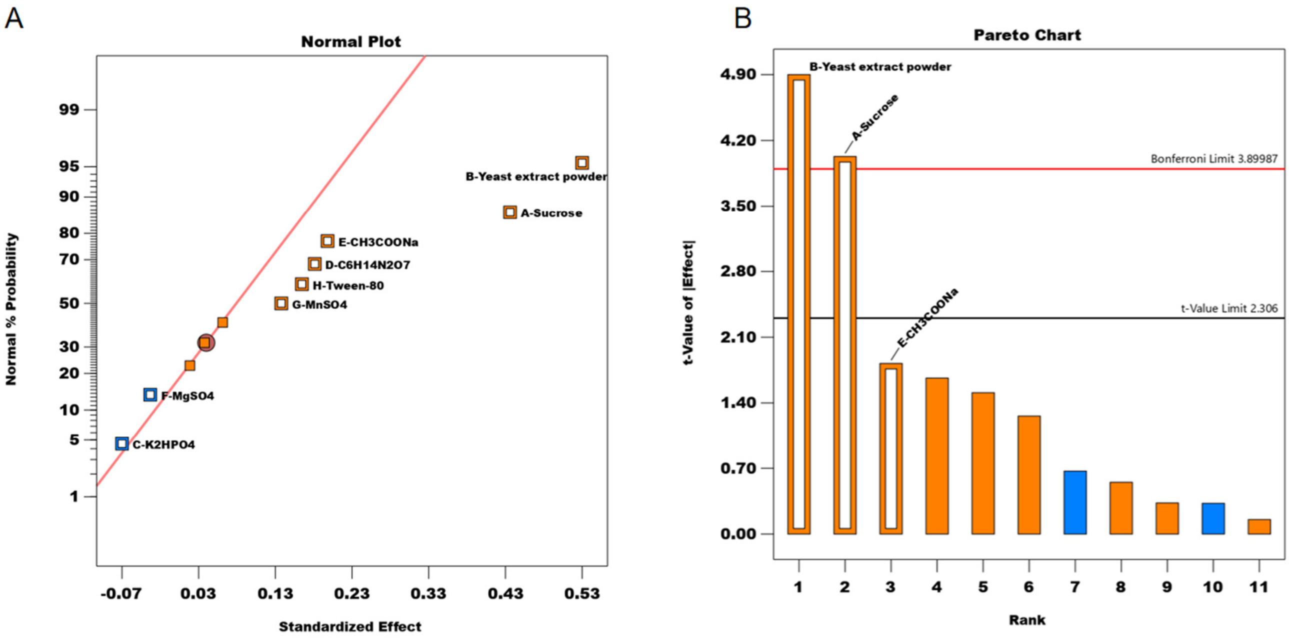

Supplementary Figure S3. Plackett-Burman analysis of the factors affecting viable cell count of *L. reuteri* W6-12. (A) Normal plot. (B) Pareto chart.

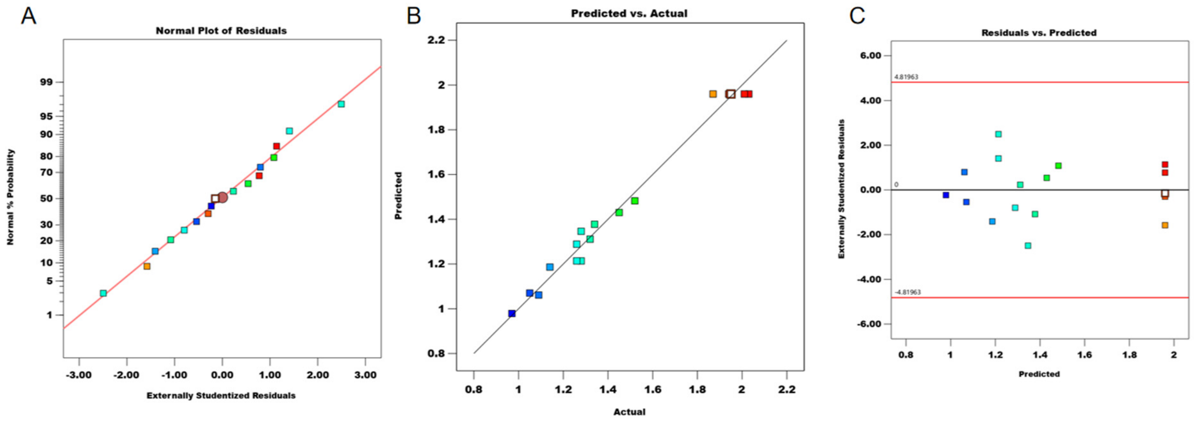

Supplementary Figure S4. Residual diagnostic plots for the Box-Behnken model of fermentation medium optimization. (A) Normal probability plot of residuals. (B) Plot of predicted vs. actual values. (C) Plot of residuals vs. predicted values.

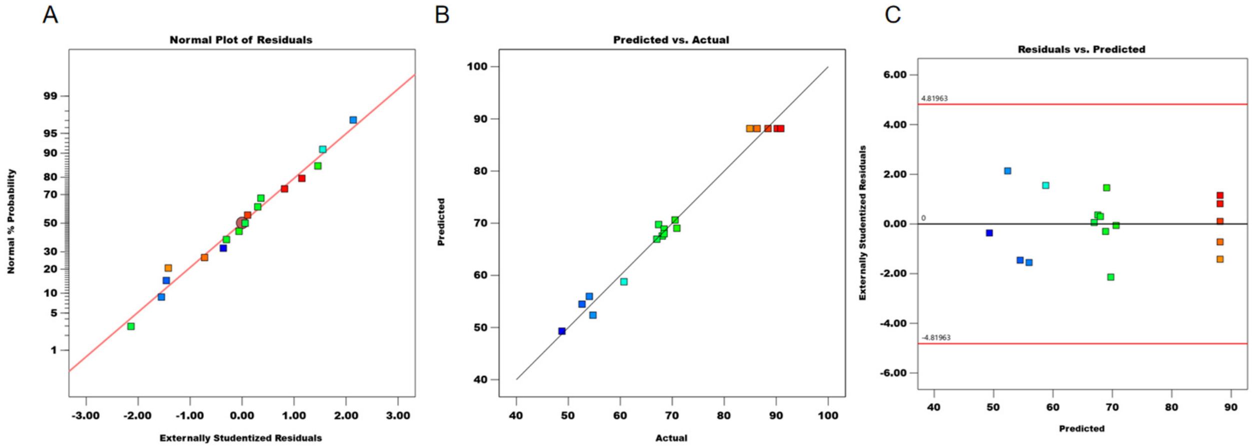

Supplementary Figure S5. Residual diagnostic plots for the Box-Behnken model of cryoprotectant formulation optimization. (A) Normal probability plot of residuals. (B) Plot of predicted vs. actual values. (C) Plot of residuals vs. predicted values.

Supplementary Table S1. Plackett-Burman experimental design and results for screening significant factors affecting viable cell count of *L. reuteri* W6-12.

Group /Factor	Optimization of Plackettt-Burman experimental conditions on culture medium (g/L)								Viable bacterial count （10 ^{9}CFU/mL）
	Sucrose	Yeast extract powder	K_2HPO_4	$C_6H_{14}N_2O_7$	CH_3COONa	$MgSO_4$	$MnSO_4$	Tween-80	
**1**	35	3	1	5	10	0.3	0.025	0.5	1.90
**2**	35	2.2	1	1	10	0.1	0.075	2.5	1.45
**3**	20	2.2	1	1	5	0.1	0.025	0.5	0.55
**4**	20	3	3	5	5	0.1	0.025	2.5	1.32
**5**	35	3	1	1	5	0.3	0.025	2.5	1.55
**6**	20	3	3	1	10	0.3	0.075	0.5	1.22
**7**	20	3	1	5	10	0.1	0.075	2.5	1.74
**8**	20	2.2	3	1	10	0.3	0.025	2.5	0.85
**9**	35	3	3	1	5	0.1	0.075	0.5	1.62
**10**	35	2.2	3	5	10	0.1	0.025	0.5	1.20
**11**	35	2.2	3	5	5	0.3	0.075	2.5	1.35
**12**	20	2.2	1	5	5	0.3	0.075	0.5	0.80

Data are mean (n = 3).

Supplementary Table S2. ANOVA for the Plackett-Burman design of fermentation medium optimization.

Source	Sum of Squares	df	Mean Square	F-value	*P*-value	
**Model**	**1.75**	8	**0.2184**	23.78	**0.0123**	**significant**
**A-Sucrose**	**0.5590**	**1**	**0.5590**	**60.85**	**0.0044**	
**B-Yeast extract powder**	**0.8269**	**1**	**0.8269**	**90.01**	**0.0025**	
**C-K_2HPO_4**	**0.0154**	**1**	**0.0154**	**1.68**	**0.2859**	
**D-$C_6H_{14}N_2O_7$**	**0.0954**	**1**	**0.0954**	**10.39**	**0.0485**	
**E-CH_3COONa**	**0.1141**	**1**	**0.1141**	**12.42**	**0.0388**	
**F-$MgSO_4$**	**0.0037**	**1**	**0.0037**	**0.4001**	**0.5720**	
**G-$MnSO_4$**	**0.0547**	**1**	**0.0547**	**5.95**	**0.0925**	
**H-Tween-80**	**0.0784**	**1**	**0.0784**	**8.54**	**0.0614**	
**Residual**	**0.0276**	**3**	**0.0092**			
**Cor Total**	**1.78**	**11**				

$P < 0.05$ indicates statistical significance.

Supplementary Table S3. ANOVA results for the response surface quadratic model of fermentation medium optimization.

Source	Sum of Squares	df	Mean Square	F-value	*P*-value	
**Model**	**2.06**	**9**	**0.2290**	**46.50**	**< 0.0001**	**significant**
**A-Sucrose**	**0.1081**	**1**	**0.1081**	**21.95**	**0.0022**	
**B-Yeast extract powder**	**0.0200**	**1**	**0.0200**	**4.06**	**0.0837**	
**C-Sodium acetate**	**0.0325**	**1**	**0.0325**	**6.60**	**0.0370**	
**AB**	**0.0182**	**1**	**0.0182**	**3.70**	**0.0958**	
**AC**	**0.0324**	**1**	**0.0324**	**6.58**	**0.0373**	
**BC**	**0.0006**	**1**	**0.0006**	**0.1269**	**0.7322**	
**A^2**	**0.3727**	**1**	**0.3727**	**75.67**	**< 0.0001**	
**B^2**	**0.8526**	**1**	**0.8526**	**173.12**	**< 0.0001**	
**C^2**	**0.4379**	**1**	**0.4379**	**88.92**	**< 0.0001**	
**Residual**	**0.0345**	**7**	**0.0049**			
**Lack of Fit**	**0.0185**	**3**	**0.0062**	**1.54**	**0.3346**	**not significant**
**Pure Error**	**0.0160**	**4**	**0.0040**			
**Cor Total**	**2.10**	**16**				

$P < 0.05$ indicates statistical significance. Lack of fit was not significant ($P = 0.3346 > 0.05$), confirming the model's goodness of fit. Significant terms are highlighted in bold.

Supplementary Table S4. ANOVA results for the response surface quadratic model of cryoprotectant formulation optimization.

Source	Sum of Squares	df	Mean Square	F-value	*P*-value	
Model	2971.02	9	330.11	44.52	< 0.0001	significant
A-L-sodium glutamate	221.55	1	221.55	29.88	0.0009	
B-Trehalose powder	119.27	1	119.27	16.09	0.0051	
C-Skim milk powder	93.64	1	93.64	12.63	0.0093	
AB	1.10	1	1.10	0.1487	0.7112	
AC	59.60	1	59.60	8.04	0.0252	
BC	44.42	1	44.42	5.99	0.0443	
A^2	974.05	1	974.05	131.37	< 0.0001	
B^2	952.08	1	952.08	128.41	< 0.0001	
C^2	270.64	1	270.64	36.50	0.0005	
Residual	51.90	7	7.41			
Lack of Fit	26.41	3	8.80	1.38	0.3696	not significant
Pure Error	25.49	4	6.37			
Cor Total	3022.92	16				

$P < 0.05$ indicates statistical significance. Lack of fit was not significant ($P = 0.3696 > 0.05$), confirming the model's goodness of fit. Significant terms are highlighted in bold.
